# *Irf8*-Regulated Genomic Responses Drive Pathological Inflammation during Cerebral Malaria

**DOI:** 10.1371/journal.ppat.1003491

**Published:** 2013-07-11

**Authors:** Joanne Berghout, David Langlais, Irena Radovanovic, Mifong Tam, John D. MacMicking, Mary M. Stevenson, Philippe Gros

**Affiliations:** 1 Department of Biochemistry and Complex Traits Group, McGill University, Montreal, Quebec, Canada; 2 McGill University Health Centre, Montreal, Quebec, Canada; 3 Section of Microbial Pathogenesis, Yale University School of Medicine, New Haven, Connecticut, United States of America; London School of Hygiene and Tropical Medicine, United Kingdom

## Abstract

Interferon Regulatory Factor 8 (IRF8) is required for development, maturation and expression of anti-microbial defenses of myeloid cells. BXH2 mice harbor a severely hypomorphic allele at *Irf8* (*Irf8^R294C^*) that causes susceptibility to infection with intracellular pathogens including *Mycobacterium tuberculosis*. We report that BXH2 are completely resistant to the development of cerebral malaria (ECM) following *Plasmodium berghei* ANKA infection. Comparative transcriptional profiling of brain RNA as well as chromatin immunoprecipitation and high-throughput sequencing (ChIP-seq) was used to identify IRF8-regulated genes whose expression is associated with pathological acute neuroinflammation. Genes increased by infection were strongly enriched for IRF8 binding sites, suggesting that IRF8 acts as a transcriptional activator in inflammatory programs. These lists were enriched for myeloid-specific pathways, including interferon responses, antigen presentation and Th1 polarizing cytokines. We show that inactivation of several of these downstream target genes (including the *Irf8* transcription partner *Irf1*) confers protection against ECM. ECM-resistance in *Irf8* and *Irf1* mutants is associated with impaired myeloid and lymphoid cells function, including production of IL12p40 and IFNγ. We note strong overlap between genes bound and regulated by IRF8 during ECM and genes regulated in the lungs of *M. tuberculosis* infected mice. This IRF8-dependent network contains several genes recently identified as risk factors in acute and chronic human inflammatory conditions. We report a common core of IRF8-bound genes forming a critical inflammatory host-response network.

## Author Summary

Cerebral malaria is a severe and often lethal complication from infection with *Plasmodium falciparum* which is driven in part by pathological host inflammatory response to parasitized red cells′ adherence in the brain microvasculature. However, the pathways that initiate and amplify this pathological neuroinflammation are not well understood. As susceptibility to cerebral malaria is variable and has been shown to be partially heritable, we have studied this from a genetic perspective using a mouse model of infection with *P. berghei* which induces experimental cerebral malaria (ECM). Here we show that mice bearing mutations in the myeloid transcription factor IRF8 and its heterodimerization partner IRF1 are completely resistant to ECM. We have identified the genes and associated networks that are activated by IRF8 during ECM. Loss-of-function mutations of several IRF8 targets are also shown to be protective. Parallel analysis of lungs infected with *Mycobacterium tuberculosis* show that IRF8-associated core pathways are also engaged during tuberculosis where they play a protective role. This contrast illustrates the balancing act required by the immune system to respond to pathogens and highlights a lynchpin role for IRF8 in both. Finally, several genes in these networks have been individually associated with chronic or acute inflammatory conditions in humans.

## Introduction

IRF8 is a member of the Interferon Regulatory Factor (IRF) family of transcription factors that plays a central role in interferon signaling, response to infection and maturation of dendritic cells (DCs) and other myeloid lineages [1], [2]. IRF8 regulates elements of constitutive gene expression in both myeloid and lymphoid cells and can also activate or suppress pathogen responsive transcription programs following exposure to type I or type II interferons, lipopolysaccharides, and a range of additional microbial products [1], [2]. Heterodimerization of IRF8 with members of the IRF (IRF1, IRF4) or ETS (PU.1) families leads to DNA binding and transcriptional regulation of target genes containing ISRE (GAAAnnGAAA) and EICE-type (GGAAnnGAAA) canonical motifs in their promoters [3]–[5].

During hematopoiesis, IRF8 promotes differentiation of myeloid progenitors towards the mononuclear phagocyte lineages (monocytes, macrophages, DCs) by acting as an antagonist of the polymorphonuclear granulocyte pathway [6]–[9]. This is accomplished through positive regulation of pro-apoptotic signals (*Cdkn2b, Nf1, Bax*), and negative regulation of pro-survival signals (*Bcl2*, *Bcl-XL*) in CD11b^+^ myeloid precursors [10]–[12]. Mice bearing either a targeted null allele [7] or a severely hypomorphic mutation (BXH2, *Irf8^R294C^*) [9] show either complete absence of all CD11c^+^CD8α^+^ dendritic cells subsets (*Irf8^−/−^*) while BXH2 mice (*Irf8^R294C^*) retain some plasmacytoid DCs (B220^+^/CD11c^low^) [13]–[15]. Furthermore, both mutants display a chronic myeloid leukemia-like phenotype dominated by expansion of Gr1^+^/CD11b^+^ immature myeloid cells [6]–[9]. Additionally, IRF8 is required for B lymphocyte lineage specification, commitment, and differentiation, including expression of certain biochemical pathways that play a key role in the specialized functions of these antigen-presenting cells (APCs) [2], [5], [16].

During infection, IRF8 activates antimicrobial defenses in myeloid cells, propagates pro-inflammatory signals and is required to amplify early immune responses by these cells. IRF8 activity is essential to express *Il12p40*, *Il12p35* and *Il18* in response to IFNγ [6], [17]–[21] and is therefore required for APC-mediated Th1 polarization of early immune responses [1], [2]. *Irf8*-deficient mice, then, display defective Th1 responses (absence of antigen specific and IFNγ producing CD4^+^ T cells) [22], enhanced Th17 responses [23], and are susceptible to *in vivo* infection with many intracellular pathogens [18], [24]–[26] including tuberculosis [22] and blood-stage malaria [27]. *Irf8*-deficient macrophages are also extremely susceptible to *ex vivo* infection by intracellular pathogens [28]–[30]. Studies using genome-wide transcript profiling, chromatin immunoprecipitation [3]–[5] and individual gene targets [1] show that IRF8 regulates multiple aspects of antimicrobial defenses in mononuclear phagocytes. These include antigen recognition and processing, phagosome maturation, production of lysosomal enzymes and other cytoplasmic microbicidal pathways.

*IRF8* mutations in humans cause pathologies remarkably similar to those observed in *Irf8* mutant mice, affecting the myeloid compartment in general and DCs in particular [31]. We have shown that homozygosity for a DNA-binding incompetent and transcriptionally inactive human *IRF8* mutant variant (*IRF8^K108E^*) is associated with severe recurrent perinatal bacterial and fungal infections, with absence of blood monocytes and DCs, and a lack of IL-12 and IFNγ production following *in vitro* stimulation of blood cells [31]. We also reported a milder autosomal dominant form of IRF8-deficiency (*IRF8^T80A^*) in two patients suffering from *Mendelian susceptibility to mycobacterial disease* (MSMD) with recurrent episodes of mycobacterial infections following perinatal vaccination with *M. bovis* BCG. These patients showed selective depletion of the CD11c^+^ CD1c^+^ DC subset and impaired production of IL-12 by circulating peripheral blood cells. The *IRF8^T80A^* variant displays negative dominance and can suppress the trans-activation potential of wild type IRF8 for known transcriptional targets such as *NOS2* and *IL-12* [31]. Interestingly, T cells raised in the absence of an intact DC compartment in patient *IRF8^K108E^* show impaired function (production of IFNγ and other cytokines in response to non-specific stimuli) [31]. Finally, recent results from genome-wide association studies (GWAS) have pointed to a role for *IRF8* in the complex genetic etiology of several human inflammatory diseases. Strong and independently replicated associations have been detected between polymorphic variants within or near *IRF8* gene for systemic lupus erythematosus [32], ulcerative colitis [33], Crohn's disease [34], [35], and multiple sclerosis (MS) [36]–[38]. Details of one study in MS patients showed that the *IRF8* susceptibility allele (rs17445836) was associated with higher expression of both *IRF8* mRNA and downstream IFNβ-responsive targets [36].

We have used an experimental model of murine cerebral malaria (ECM) induced by infection with *Plasmodium berghei* ANKA (PbA) to investigate the role of *Irf8* in pathological inflammation. In this model, adherence of PbA-infected erythrocytes to brain microvasculature leads to acute and rapidly fatal neuroinflammation. Symptoms such as tremors, ataxia and seizures appear between d5 and d8 in susceptible mice, progressing to morbidity and death within hours. *Irf8*-deficient BXH2 mice (*Irf8^R294C^*) did not develop any neurological symptoms and were found to be completely resistant to PbA-induced ECM. Comparative transcript profiling of PbA-infected wild-type C57BL/6 (B6) and BXH2 mice, together with IRF8 chromatin immunoprecipitation coupled to high-throughput DNA sequencing (ChIP-seq) have identified a list of key *Irf8* targets whose expression is associated with acute ECM-associated neuroinflammation. This list has substantial overlap with genes activated in mouse lungs following infection with *M. tuberculosis* (Mtb), suggesting a shared core inflammatory response to infection that is protective against Mtb infection but deleterious in ECM. These studies identify IRF8 as a key regulator of acute neuroinflammation during ECM and a major inflammatory mediator.

## Results

### Inactivation of *Irf8* causes resistance to cerebral malaria

**Figure 1:**
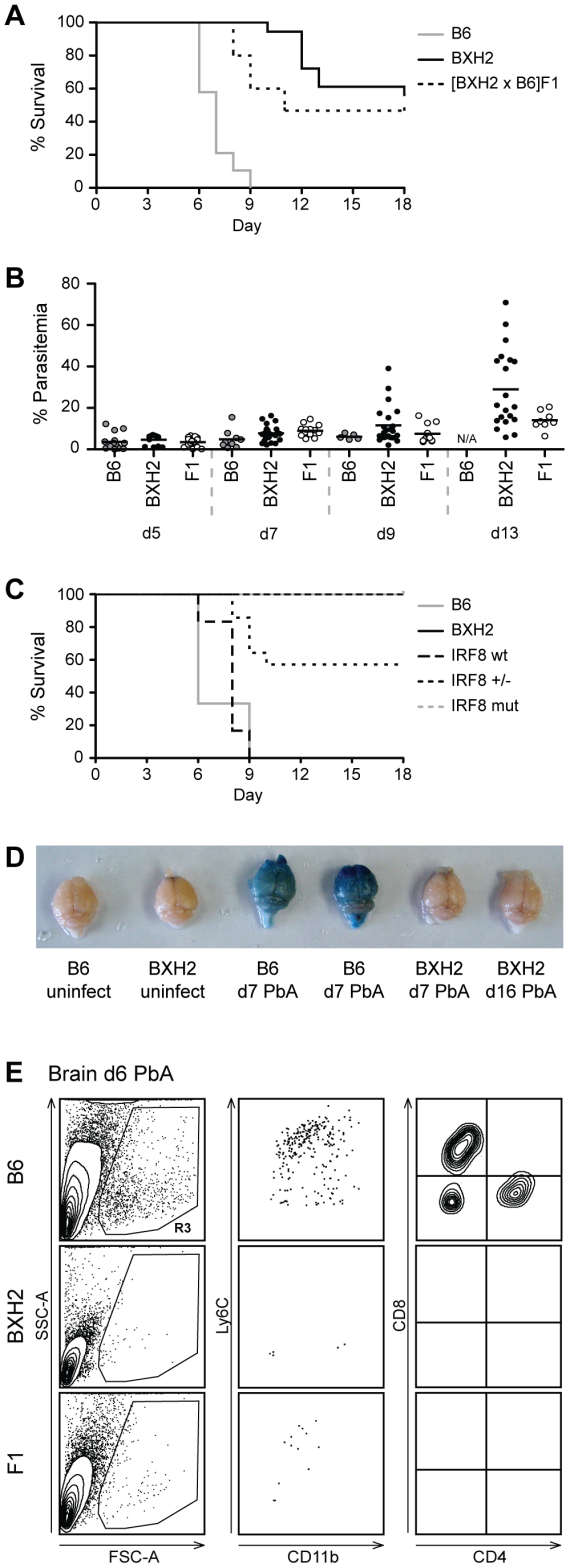
BXH2 mice do not develop cerebral malaria following *Plasmodium berghei* infection. (A) Survival plots of *Plasmodium berghei* (PbA) infected BXH2 mice (n = 18), heterozygous [BXH2×B6]F1 offspring (n = 15), and ECM-susceptible B6 (n = 19) parental controls. Shown are the combined results of four experiments. (B) Blood parasitemia levels during infection for mice in Panel A following infection with PbA. (C) *Irf8* genotype-specific survival curves for 24 [BXH2×B6]F2 mice along with parental controls. (D) Qualitative comparison of representative Evans blue dyed brains from uninfected and infected B6 and BXH2 mice indicating breakdown of the blood-brain barrier in infected B6 (d7 PbA), but not BXH2 (d7 PbA or d16 PbA) mice. (E) Perfused brains from ECM-susceptible B6 and ECM-resistant BXH2 and [BXH2×B6]F1 mice (n = 2 to 4) were collected six days following infection with PbA. Infiltrating leukocytes were enriched by Percoll gradient and stained with CD45, Ly6C and CD11b, or CD45, TCRβ, CD4 and CD8 antibodies. The presence of myeloid and lymphoid infiltrates is observed in the brain of B6 mice compared to BXH2 or F1. Gate R3 denotes infiltrating cells gated by side scatter (SSC-A) and forward scatter (FSC-A).

BXH2 is a recombinant inbred mouse strain derived from B6 and C3H/HeJ (C3H) parents which displays a myeloid defect in the form of immature myeloid hyperplasia and susceptibility to multiple infections. We have previously used high resolution linkage analysis, positional cloning and candidate gene sequencing to demonstrate that myeloid hyperplasia and susceptibility to infections are caused by a severely deleterious hypomorphic allele at *Irf8* (*Irf8^R294C^*) that spontaneously arose during the breeding of this strain [9], [12]. To assess the contribution of *Irf8* to pathological inflammation, we infected BXH2 mice with PbA parasites, the murine agent of experimental cerebral malaria (ECM). Parasite replication in the blood, appearance of neurological symptoms and overall survival were recorded over 18 days (Figure 1). While all B6 mice developed ECM and succumbed by d9, BXH2 mice were completely resistant to the ECM phase, succumbing later to hyperanemia caused by uncontrolled blood-stage replication of the parasite (Figures 1A and 1B). [BXH2×B6]F1 mice showed significant resistance to PbA-induced ECM when compared to susceptible B6 parental control (p<0.0001), with approximately 50% of the animals surviving past d9 (Figure 1A). Additional phenotyping of a small group of segregating [BXH2×B6]F2 mice (n = 24) identified ECM-resistance only in mice either homozygote or heterozygote for the *Irf8^R294C^* allele, confirming that the protective effect we observed is due to the *Irf8^R294C^* mutation with minimal or no contribution of the mixed B6/C3H genetic background of BXH2 (Figure 1C). These data show that a) partial or complete loss of IRF8 function protects mice against lethality in an ECM-associated neuroinflammation model, and b) that the ECM-protective effect of the *Irf8^R294C^* mutation is inherited in a co-dominant fashion. Resistance to ECM in BXH2 mice was not associated with decreased parasite burden, as B6, BXH2 and [BXH2×B6]F1 mice showed similar levels of circulating blood parasitemia at d5, d7 and d9 post-infection (p>0.1)(Figure 1B). However, as the infection progressed, some BXH2 mice developed extremely high levels of blood parasitemia between d12-d21 in sharp contrast to any surviving controls or [BXH2×B6]F1s. This high parasitemia, rather than cerebral inflammation, was responsible for the observed mortality.

Lethal ECM in PbA-infected mice is associated with endothelial dysfunction, including loss of integrity of the blood brain barrier (BBB) [39]–[41]. Using an Evans Blue dye extravasation assay (Figure 1D), PbA-infected B6 mice display obvious BBB permeability by d7, indicated by the blue color, while infected BXH2 mice retained BBB integrity both early (d7) and late (d16) during infection. The BBB integrity was also assessed by flow cytometry cellular profiling of perfused brains from day 6 infected mice. In B6 brains, ECM was associated with presence of both CD4^+^ and CD8^+^ lymphocytes, as well as CD11b^+^/Ly6C^+^ granulocytes and monocytes (Figure 1E). Interestingly and despite the immature myeloid hyperplasia in peripheral myeloid and lymphoid organs, infiltration of T cells and myeloid cells in the brain was not seen in PbA-infected BXH2, nor was it seen in [BXH2×B6]F1 (Figure 1E). Together, these results indicate that ECM susceptibility in B6 is associated with infiltration of myeloid and lymphoid cells at the site of pathology. Such infiltration is not seen in ECM-resistant mice that are either homozygous (BXH2) or heterozygous ([BXH2×B6]F1) for *Irf8^R294C^*.

These results demonstrate that although IRF8 dysfunction is protective against ECM, functional IRF8 is required to control blood stage replication of PbA late in infection. Partial IRF8 activity in [BXH2×B6]F1 is sufficient to protect against high blood-stage replication.

### Differential transcript profiles associated with *Irf8*-dependent resistance to cerebral malaria

**Figure 2:**
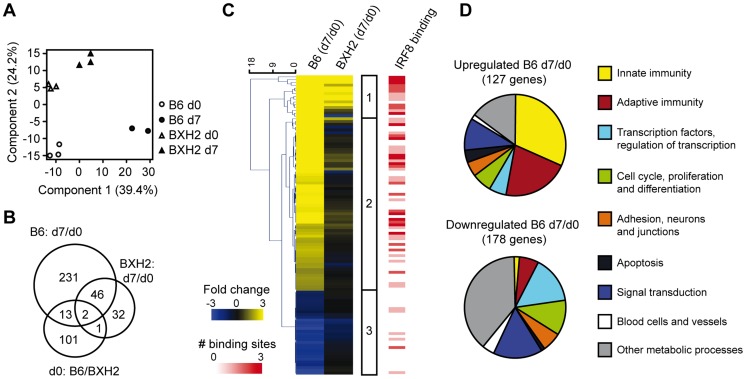
Transcript profiling of PbA infected B6 and BXH2 brains reveals strain and infection specific differences. (A) Unsupervised principal components analysis clusters samples according to mouse strain and infection status. (B) Intersection of gene lists generated by pairwise comparisons between infected and uninfected B6 and BXH2 transcript profiles. (C) Euclidean clustered heat map of transcripts regulated in both a strain and infection specific manner (two-factor ANOVA, p_adj_-interaction<0.05) illustrated as infection-induced fold change in each strain (d7/d0). Each row represents a unique gene, and in cases where two or more transcript probes for a gene were significant, the average fold change was used. Differential expression patterns clustered into three groups with Group 1 genes being up-regulated by infection in both strains, Group 2 genes up-regulated by infection in B6 mice and unresponsive in BXH2 and Group 3 genes down-regulated by infection, typically more so in B6 than BXH2. See Table S1 for details. Red shaded heat map indicates the presence of one or more IRF8 binding sites within 20 kb of the gene transcription start site as determined by ChIP-seq. (D) Gene ontology for transcripts differentially regulated by infection during ECM pathology in B6 mice (as determined by B6 d7/d0 pairwise analysis, p_adj_<0.05). Up-regulated genes are predominantly involved in innate and adaptive immunity processes, while down-regulated genes are not, and rather include a variety of homeostatic biological and metabolic processes.

To gain further insight into the genes, proteins and pathways that play a role during pathological neuroinflammation, and identify those whose expression is regulated by *Irf8*, we used transcript profiling of BXH2 and B6 brains either prior to or during PbA-infection. Principal components analysis (PCA) clustered the samples along two axes: component 1, which explained 39.4% of the variance and was associated with infection status (infection component), and component 2, associated with mouse strain (genetic component), which explained 24.4% of the variance (Figure 2A). PCA also indicates that PbA infection had a much stronger impact on transcriptional profiles in B6 mice than in BXH2, with the B6 d7 infected samples forming a remote out-group. In contrast, the BXH2 d7 cluster was only moderately shifted by infection and remained much closer to the BXH2 d0 group (compared to B6 d0 vs. B6 d7) indicating more modest response to infection by BXH2.

We used paired t-tests to assess the infection-induced transcriptional responses in both B6 and BXH2 mice as a way to extract gene lists relevant to ECM susceptibility. As suggested by the PCA, B6 response to infection was robust, with 292 genes showing statistically significant differences in expression (Figure 2B, d0 vs. d7; fold change ≥2, p_adj_<0.05). On the other hand, response to infection in BXH2 was more modest with 81 genes reaching statistical significance. More than half of the genes (n = 48) regulated by infection in BXH2 were common to the B6 set and may indicate *Irf8*-independent regulatory mechanisms. This analysis also identified a set of 117 genes that show significantly different levels of expression in B6 and BXH2 mice prior to infection. Only ∼10% of these genes (n = 16) were further significantly modulated by PbA infection (Figure 2B). Importantly, this analysis further identified a key subset of 231 genes that were specifically regulated in B6, but not BXH2, mice during infection, therefore associated with pathology.

We also performed a two-factor ANOVA test accounting for both differences in basal level of gene expression in the brain (B6 vs. BXH2 at day 0), and infection-induced transcriptional response to PbA (Figure 2C). This identified a total of 107 genes (123 probes; fold change ≥2, p_adj_<0.05) that were strongly regulated by infection in an *Irf8*-dependent fashion (p_adj_-interaction <0.05) (Figure 2C, Table S1). Euclidean hierarchical clustering of this gene list identified three major categories of transcripts. Group 1 genes (n = 15) expression levels were increased by infection in both strains (more pronounced in B6 than BXH2), group 2 genes were increased by infection in B6 mice but not significantly induced in BXH2 (n = 62) and group 3 genes (n = 30) expression was reduced in infected mice (stronger repression in B6 compared to BXH2). Using the online Database for Annotation and Integrated Discovery (DAVID) tool to examine the list of genes regulated by infection in B6 mouse brains indicated substantial enrichment for immune response (4.4-fold enrichment above Illumina WG-6 v2.0 chip background, p_adj_ = 3.3×10^−12^), antigen processing and presentation (11.0-fold enrichment, p_adj_ = 1.4×10^−9^), defense response (3.8-fold enrichment, p_adj_ = 1.7×10^−8^), chemotaxis (5.2-fold enrichment, p_adj_ = 3.4×10^−3^) and inflammatory response (3.6-fold enrichment, p_adj_ = 3.7×10^−3^). Increased genes on these lists include potent pro-inflammatory chemokines that recruit myeloid and lymphoid cells to the site of infection and/or tissue injury such as *Cxcl9, Cxcl10, Ccl4*, and *Ccl12*; myeloid cell receptors associated with phagocytosis of microbes (*Fcgr3*, *Fcgr4*) and maturation of phagosomes (small GTPases *Igtp, Irgm1, Gbp2, Gbp3*); IRF8's hetero-dimerization partner (*Irf1*), and early type I interferon response (*Oasl2, Ifit3*). Genes under these categories were expressed more highly in B6 than in BXH2, consistent with the notion that resistance to ECM-associated neuroinflammation in BXH2 is linked to reduced *Irf8*-dependent inflammatory and innate immune responses, with a strong involvement of the myeloid compartment.

Organizing the list of genes associated with ECM pathology (B6 d7/d0 pairwise) using Ingenuity Pathway Analysis revealed enrichment for several significant biological networks. The top-scoring network included down-regulation of the hematological response and increase of intercellular signaling (Figure S1A). Several immune cell chemoattractants (*Ccl4*, *Ccl7*, *Cxcl9*) are featured in this network, suggesting recruitment of monocytes, NK cells and T-cells to the site of brain inflammation. Very little modulation of either of these pathways is seen in BXH2 during infection, with most components not making the 2 fold change threshold (Figure S1B). The second network highlights significant increase of interferon-responsive genes and inflammatory mediators, with type 1 interferon, *Irf7* and *Stat1* regulation occupying highly connected nodes (Figure S1C). Most of these inflammatory processes are increased to a lesser degree in BXH2 mice (Figure S1D and details in Table S1). The third network focuses on antigen processing and presentation with immunoglobulins and Fc receptors increased at the center of the B6 infection-responsive image (Figure S1E). Similar to what was seen in network 1, significant increase of these genes was largely missing from the BXH2 response (Figure S1F).

### Chromatin immunoprecipitation and sequencing (ChIP-seq) highlights IRF8-bound genes activated during acute neuroinflammation

**Figure 3:**
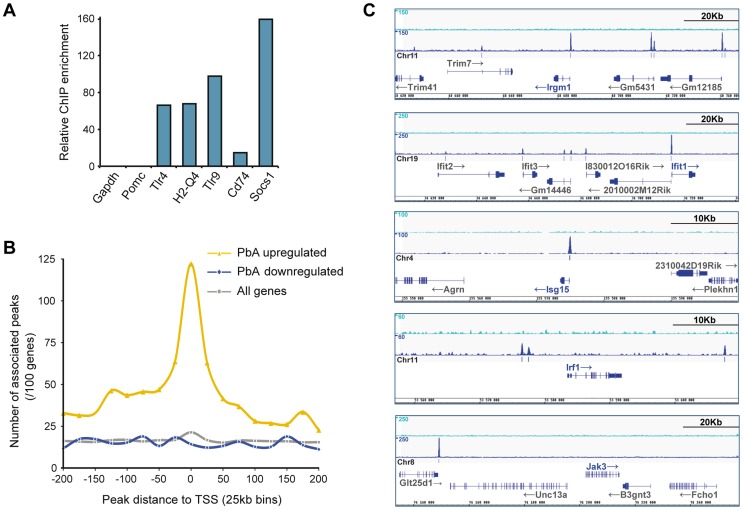
Genes up-regulated during ECM pathology in B6 mice are significantly enriched for IRF8 binding sites. (A) Quantitative PCR was used to validate ChIP results using known binding targets of IRF8. Targets were highly enriched in IRF8-immunoprecipitated DNA when compared to control IgG preparations. Representative data from one of five independent experiments is shown. (B) The list of genes regulated by infection in B6 mice (d7/d0 pairwise) was interrogated for IRF8 binding sites within 225 kb of their transcription start site. The graph represents the abundance of IRF8 binding sites in each 25 kb segment. (C) IRF8 and control IgG ChIP-seq sequence reads were mapped to the mouse genome and significant IRF8 binding sites were identified. Light blue (top) track indicates non-specific (IgG) sequencing profile and dark blue track (below) displays IRF8 binding sites. Genes were considered to have an IRF8 binding site if a peak was found within 20 kb of the transcription start site.

Since total brain RNA was used in our studies, genes differentially regulated in response to infection in an *Irf8*-dependent fashion may represent direct transcriptional targets of IRF8 or may be secondary targets corresponding to markers of cell populations differentially recruited to the site of infection in B6 and BXH2 mice. To distinguish between these possibilities, we mapped genome-wide IRF8 binding sites using ChIP-seq. The resulting sequence reads were mapped to mouse mm9 genome assembly and IRF8 binding peaks were identified using MACS peaks finding algorithm [42]. In order to validate ChIP-seq results, we confirmed IRF8 recruitment on known target sites [3] by independent ChIP-qPCR experiments (Figure 3A). IRF8-bound genes were identified as those containing a binding peak within a 20 kb window from their transcriptional start site (TSS). The list of IRF8-bound genes by ChIP-seq was intersected with the list of genes regulated by PbA in a strain, infection and *Irf8*-associated fashion from the two-factor ANOVA analysis (Figure 2C, Table S1). This intersection revealed a strong enrichment of IRF8 binding sites in genes increased during infection, with IRF8 binding sites detected in 85% of Group 1 genes (13/15) and 50% of Group 2 genes (31/62) (Figure 2C, Table S1). Differentially down-regulated genes did not show any enrichment with only 13% (4/30) of Group 3 genes containing IRF8 peaks, lower than background peak association (21% of all genes represented on the Illumina array; Figure 3B). These results strongly suggest that during neuroinflammation, IRF8 functions as a direct transcriptional activator of multiple genes coding for key pro-inflammatory pathways.

To identify IRF8-bound genes associated with ECM neuropathology, we queried the list of all genes whose expression is regulated by infection in *Irf8*-competent B6 mice for the presence of IRF8 binding sites (pair-wise comparison of B6 d7/d0). This analysis (Figure 3B) showed very strong enrichment for IRF8 binding sites in the vicinity of genes increased by infection, with 74% of increased genes (92/125; p<0.0001, Fisher's Exact test) bearing one or more IRF8 binding sites within 20 kb of the TSS (Figure 3C for ChIP-seq profile examples and complete list in Table S2). Genes decreased in response to infection did not show enrichment above background (Figure 3B and Table S2). Using the AmiGO gene ontology annotation tool [43], this list (Figure 2D and see details in Table S3) was found to contain numerous genes involved in inflammatory and innate immune responses, a finding most pronounced in the subset (74%) of genes with IRF8 binding sites. This included inflammatory cytokines and chemokines involved in chemotaxis of myeloid and lymphoid cell types to the sites of infection (*Ccl4, Ccl5, Ccl7, Ccl12, Cxcl9, Cxcl10*), early innate immune recognition and responses (*Nlrc5, Ifi205*), response to viral infections (*Oasl2, Mx2, Oas1g*), type I interferon responsive genes and pathways (*Ifit2, Ifit3, Isg15, Rsad2*), antigen capture (*C1q, C4b, Fcerg1*), phagosome maturation (*Irgm1, Irgm2, Igtp, Gbp2, Gbp3*), antigen processing (*Tap1, Tap2*) and Class I and Class II MHC-dependent antigen presentation in myeloid cells (*B2m, H2-Ab1, H2-D, H2-K, H2-L, H2-Q, H2-T22* ). Other IRF family members implicated in early response to antigenic stimuli or danger signals (*Irf1, Irf7, Irf9*) were also induced (Table S3). These IRF8-regulated pro-inflammatory pathways appear linked primarily to the myeloid cellular compartment.

### Activation of a common Irf8 transcriptome in cerebral malaria and during pulmonary tuberculosis

In both humans [31] and mice [22], mutations in *IRF8* cause susceptibility to mycobacterial infections. Thus, we compared the list of genes regulated by infection in B6 brains during PbA cerebral malaria (d7/d0) with the list of genes contributing to the protective response in the lungs of *M. tuberculosis* infected B6 mice (d30/d0) [22] (Table S2). B6 and BXH2 mice have opposite phenotypes in the two disease models, with B6 being susceptible to ECM and resistant to *M. tuberculosis*, while BXH2 succumb rapidly to pulmonary tuberculosis [22]. Strikingly, out of the 123 genes increased more than 2-fold during ECM, 66 followed the same trend during *M. tuberculosis* infection (p<0.0001, Fisher's Exact Test). There was minimal overlap in the down-regulated genes (21 *M. tuberculosis*-regulated genes overlapping with the 170 PbA-regulated, including 6 in the opposing direction). The overwhelming majority (80%) of genes increased during both infections contained at least one IRF8 binding site, again highlighting IRF8 as having a central role during inflammation and host response to infections (Table S2).

**Figure 4:**
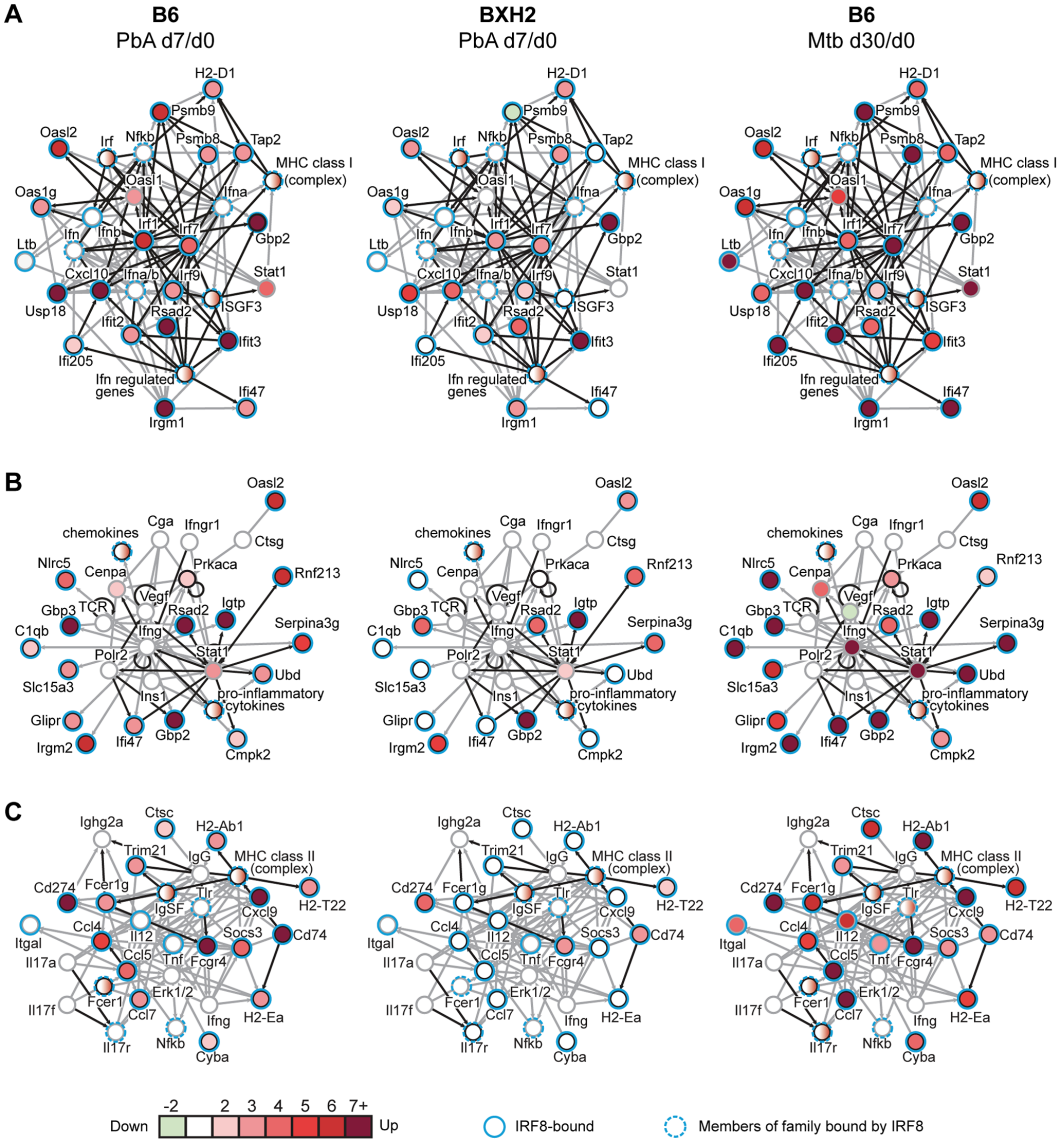
IRF8-regulated pro-inflammatory networks commonly activated during cerebral malaria and pulmonary tuberculosis. Networks were generated for the 53 genes up-regulated in B6 mice by both infections which also possess an IRF8 binding site within 20 kb of the TSS (see Table S2 for details). (A) Top scoring network highlighting IRF signaling and MHC class I antigen presentation with gene circles colored according to fold change during PbA infection in B6 d7/d0 (left), BXH2 d7/d0 (center) and *M. tuberculosis* infection in B6 d30/d0 (right). (B) Second top scoring network highlights *Ifng* and *Stat1* signaling. (C) Third top scoring network highlights MHC Class II and Fc receptors. Genes included in the list of 53 are represented by black circles, while networked genes added by the software are outlined in gray. IRF8-bound genes are outlined in light blue. Direct (black) and indirect (gray) connections between genes are shown by arrows.

To visualize the core networks engaged in this common IRF8-regulated host response, we analyzed the 53 genes containing IRF8 binding sites, and which were increased during both ECM and in pulmonary tuberculosis (Figure 4 and see Table S2 for details). Several networks with a clear focus on type 1 and type 2 interferon pathways characteristic of a pro-inflammatory innate immune response were detected. All these networks are clearly less activated in PbA infected BXH2 mice. One obvious network (Figure 4A) includes three Irfs (Irf1, Irf7, Irf9) and other hallmarks of type 1 interferon and MHC class 1 response. A second network (Figure 4B) centers on IFNγ/STAT1 signaling, with interferon-inducible GTPases, pro-inflammatory cytokines and chemokines forming the downstream response. A third, classically myeloid network (Figure 4C) features Fc-γ receptors, immunoglobulins and MHC class 2 molecules, as well as T-cell activating IL-12, CCL4 and CCL5. Across all networks, the most highly connected genes were *Irf1*, *Irf7*, *Ifng*, and *Stat1*, consistent with a key role for IRF8 in regulating pro-inflammatory innate immune responses in myeloid cells.

### Validation of Irf8 targets as key mediators of neuroinflammation during cerebral malaria

**Figure 5:**
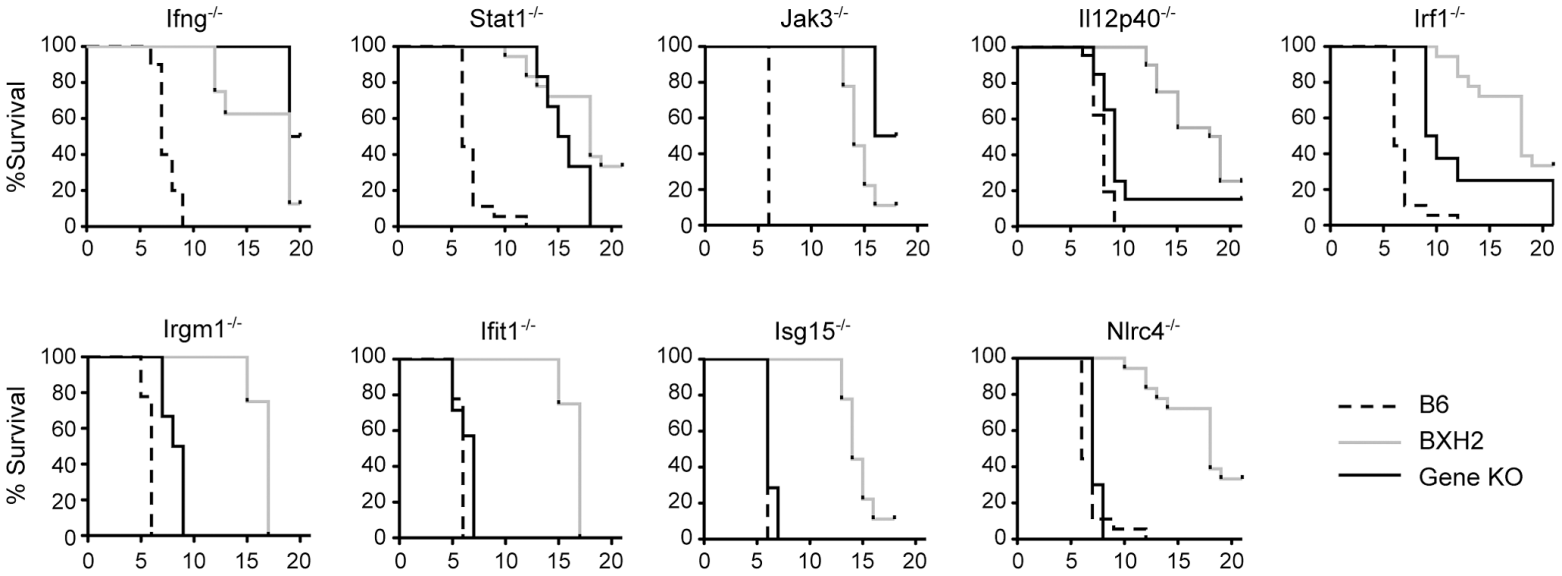
Effect of deletion of *Ifng*, *Stat1*, *Jak3*, *Irf1*, *Irgm1*, ***Il12p40***, *Ifit1*, *Isg15* and *Nlrc4* on susceptibility to PbA induced cerebral malaria. Control and mutant mice were infected with 10^6^ *P. berghei* parasites and survival was monitored. Cerebral malaria susceptible mice succumbed between d5 and d10 post-infection with neurological symptoms, while no mice that survived longer than 13 days developed signs of ECM and these were categorized as resistant. Infection specific B6 and BXH2 controls (n>5 per infection) are plotted alongside each mutant strain.

To validate the role of several identified IRF8 targets and associated pathways in pathological neuroinflammation, we phenotyped targeted knock-out mice for several of these loci. These included infection-regulated genes bearing IRF8 binding sites (*Irf1*, *Ifit1*, *Isg15*, *Nlrc4*, *Il12p40* and *Irgm1*; examples of IRF8 ChIP-seq profiles are shown in Figure 3C), and genes known to play key roles in early innate immune response (*Ifng, Jak3, Stat1, Il12p40*). Results from these experiments (Figure 5) show that *Ifng^−/−^*, *Jak3^−/−^* and *Stat1* ^−/−^ mutant mice were completely resistant to ECM following PbA infection, highlighting key roles for these molecules in the progression or amplification of the pathological inflammatory response and supporting their central positions in the network models (Figures S1C, 4A and 4B) [44], [45]. Loss of *Irf1* [46], *IL12p40* [47] and *Irgm1* delayed appearance of neurological symptoms and prolonged survival of PbA-infected mice but did not ultimately confer complete protection (Figure 5). These results validate that certain of IRF8's transcriptional targets during PbA infection in lymphoid and myeloid cells play critical downstream roles in pathology. On the other hand, *Ifit1^−/−^*, *Isg15^−/−^* and *Nlrc4* ^−/−^ mutant mice remained susceptible to PbA-induced ECM, suggesting that although these proteins may play important roles in neuroinflammation, their inactivation is not sufficient to induce protection.

### ECM resistance in IRF1 and IRF8 deficient mice is associated with altered IL12p40 and IFNγ production

**Figure 6:**
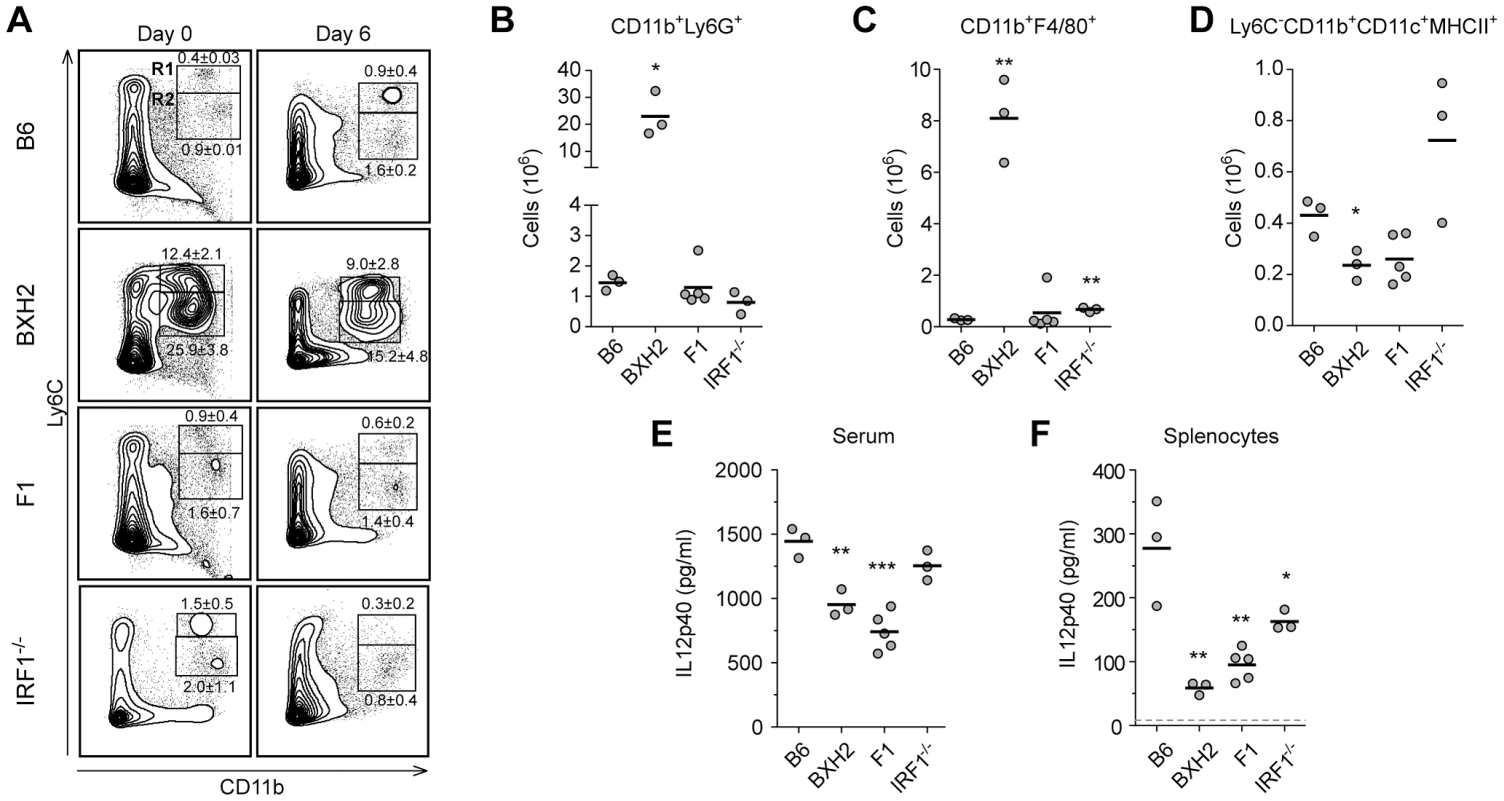
Characterization of the myeloid compartment in PbA-infected B6, BXH2, [BXH2×B6]F1 hybrids and *Irf1^−/−^*. Spleens from B6, BXH2, [BXH2×B6]F1 and IRF1^−/−^ mice were harvested prior to or six days following PbA infection, and processed by flow cytometry. 2×10^6^ splenocytes were stained with CD45, CD11b, Ly6C, Ly6G, F4/80, CD11c and MHCII, and representative cellular profiles are shown for each strain (A). The numbers within contour plots refer to gates R1 (monocytes) or R2 (granulocytes) and are reported as mean ± SD (gated as percentages of CD45^+^ cells). Absolute numbers of day 6 PbA-infected mice are shown for (B) CD11b^+^Ly6G^+^ and (C) CD11b^+^F4/80^+^ populations, indicating an expansion of the myeloid lineage in the BXH2 strain, compared to B6, [BXH2×B6]F1 and IRF1^−/−^ animals. Reduced numbers of myeloid dendritic cells (D) along with lower serum IL12p40 levels (E) are noted in ECM-resistant BXH2 and [BXH2×B6]F1 compared to ECM-susceptible B6 mice. (F) Levels of secreted IL12p40 were determined in culture supernatants from splenocytes of infected mice. Dashed gray line represents IL12p40 detection limit. Differences were considered significant when p<0.05 and calculated compared to the B6 strain (Student's t-test: *p<0.05, **p<0.01, ***p<0.001).

**Figure 7:**
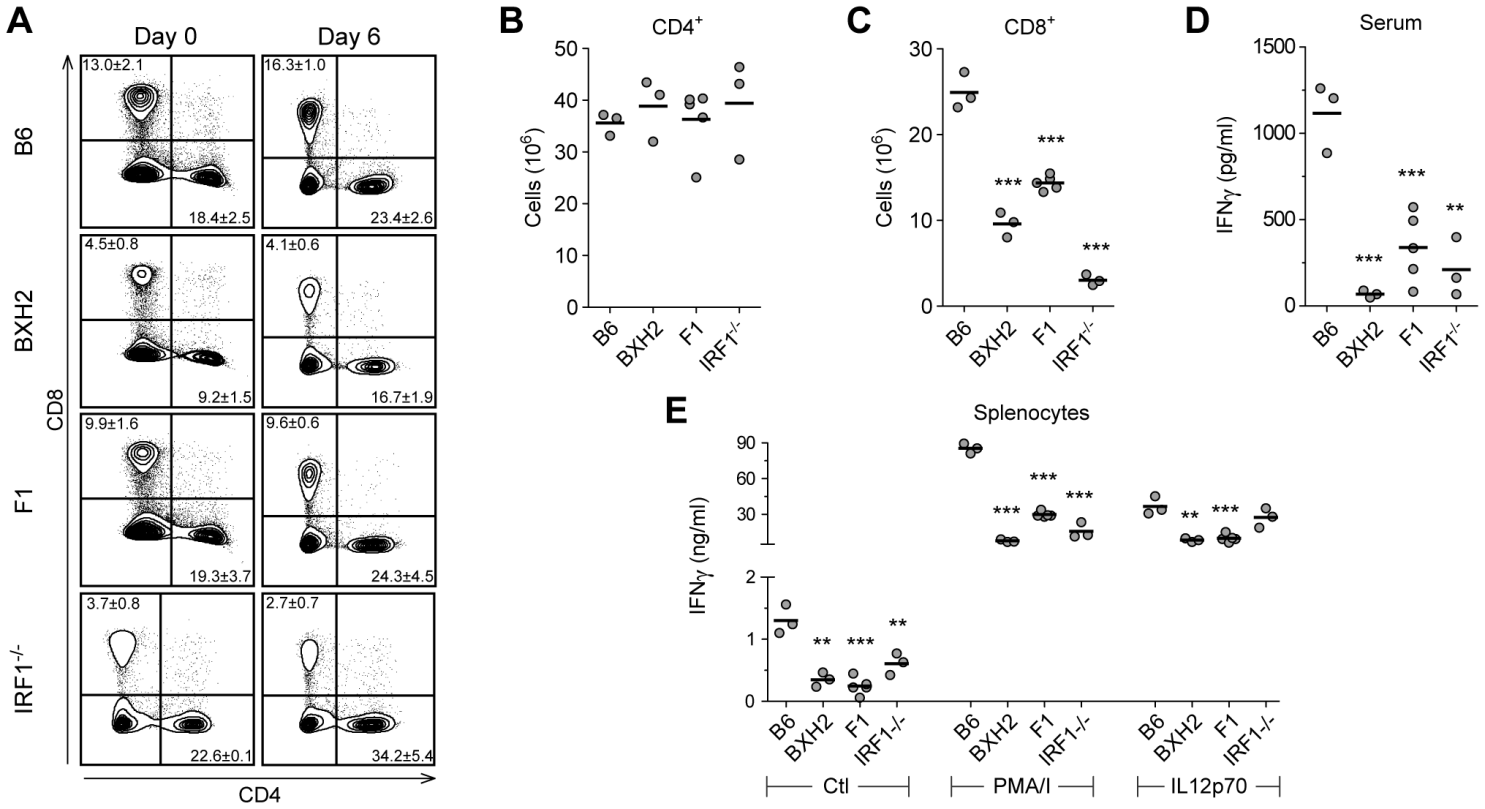
Characterization of the lymphoid compartment in PbA-infected B6, BXH2, [BXH2×B6]F1 and *Irf1^−/−^* mice. Characterization of the lymphoid compartment was carried out as described in the legend of Figure 6. Splenocytes were stained with CD45, TCRβ, CD4 and CD8 antibodies and representative cellular profiles are shown for each strain (A), where numbers indicate mean ± SD (gated as percentages of CD45^+^ cells). Absolute numbers are indicated in dot plots for total spleen CD4^+^ cells (B) and CD8^+^ cells (C). (D) Serum IFNγ levels were significantly lower in ECM-resistant BXH2, [BXH2×B6]F1 and *Irf1^−/−^* mice. (E) IFNγ production was assayed *in vitro* in culture supernatants from infected mice with or without stimulation with PMA/Ionomycin or with IL12p70. The p-values were calculated relative to B6 controls with Student's t-test (**p<0.01, ***p<0.001).

To clarify the immunological basis of ECM resistance in BXH2, and in [BXH2×B6]F1, cellular immunophenotyping of spleen cells was carried out, both at steady state (d0) and 6 days following infection with PbA, immediately preceding the appearance of neurological symptoms (Figure 6–7). In uninfected spleens, BXH2 were characterized by a dramatic expansion of CD11b^+^/Ly6C^+^ immature myeloid cells comprised of monocyte-like F4/80^+^ (Figure 6A Day 0 - Gate R1 and Figure S2A Gate R1) and granulocyte-like Ly6G^+^ cells (Figure 6A Day 0 – Gate R2 and Figure S2A Gate R2), which resulted in reduced proportions of CD4^+^ and CD8^+^ T lymphocytes (Figure 7A Day 0). In contrast, lymphoid and myeloid cell populations are highly similar in spleens of ECM-susceptible B6 and ECM-resistant [BXH2×B6]F1 mice prior to PbA infection (Figure 6A and 7A). During *P. berghei* infection, we noted that ECM-resistance in BXH2 and [BXH2×B6]F1 compared to B6 controls was associated with reduced numbers of splenic myeloid DCs (Fig. 6D; Ly6C^−^CD11b^+^CD11c^+^MHCII^+^), which was concomitant to reduced but not absent serum levels of IL12p40 (Figure 6E) and reduced production of IL12p40 by spleen cells *ex vivo* 6 days post-infection (Figure 6F). We also noted that during infection, the ECM-protective effect of the *Irf8* mutation is phenotypically expressed in the lymphoid compartment of BXH2 and [BXH2×B6]F1. Indeed, at day 6 post-infection ECM-resistant BXH2 and F1 mice did not show the significant increase in CD8^+^ T cells that was detected in ECM-sensitive B6 mice (2× increase; Figure 7C and S3), while CD4^+^ T cell numbers were increased in all groups by the same factor. Together, these changes were accompanied by a significantly reduced production of IFNγ (6 days post-infection) by splenocytes *ex vivo* measured under non-stimulated conditions (4–5×decrease), or in response to PMA/ionomycin, or to IL12p70 treatment (Figure 7E). We also noted a 16 and 3 fold reduction in the level of serum IFNγ in infected ECM-resistant BXH2 and F1 mice compared to B6 controls, respectively (Figure 7D). These studies suggest that heterozygosity and homozygosity for a loss of function allele at *Irf8* (*Irf8^R294C^*) impacts the activity of both myeloid cells and T cells during *P. berghei* infection (resulting in anergy of CD8^+^ T cells).

Irf1 cooperates with Irf8 to regulate gene expression [1], and *Irf1^−/−^* mice are resistant to ECM (Figure 5). Therefore, we also conducted immunophenotyping studies of *Irf1^−/−^* mice before (d0) and during *P. berghei* infection. As previously described [48], *Irf1^−/−^* mice display defective lymphocyte maturation, with normal CD4^+^ and greatly reduced CD8^+^ T cells numbers (Figure 7A–C). Following *P. berghei* infection, *Irf1^−/−^* mice exhibit slight reduction in IL12p40 serum level (*p* = 0.0585) and a significant decrease in IL12p40 production by splenocytes (Figure 6E–F). Altered IL12p40 production and reduced CD8^+^ T cell numbers (Figure 7A, C) is associated with severe reduction in IFNγ production, both in serum and splenocytes cultures supernatants (Figure 7D–E). *Irf1^−/−^* splenocytes produce much less IFNγ than ECM-susceptible B6 mice in response to external secondary trigger such as PMA/ionomycin (Figure 7E). Taken together, the combined analysis of *Irf1* and *Irf8* mutant mice associates reduced pro-inflammatory cytokines production (IL12p40 and IFNγ) to resistance to ECM.

## Discussion

The demonstrated role of IRF8 in the ontogeny of the myeloid lineage, its known role in defense against infectious pathogens and the growing body of evidence from GWAS in humans linking IRF8 variants to chronic inflammatory conditions such as multiple sclerosis [36], systemic lupus erythematosus [32] and inflammatory bowel disease [34], [35], [49] prompted us to investigate a possible role for IRF8 in acute pathological inflammatory reactions. To do so, we used a mouse model of acute cerebral malaria encephalitis caused by infection with PbA, which involves lethal neuroinflammation associated with recruitment of inflammatory mononuclear and polymorphonuclear leukocytes, and loss of integrity of the blood brain barrier (BBB). We found that the severe loss of *Irf8* function in BXH2 mice completely protects against this pathology, preventing the development of neurological symptoms and prolonging survival post-infection. Interestingly, the protective effect was inherited in a co-dominant fashion as 50% of *Irf8* ^R294C/+^ F1 heterozygotes survived through the cerebral phase when infected with PbA (Figure 1A–B). These finding establish that *Irf8* is critical to the development of acute lethal neuroinflammation and further implicate *Irf8* as a major regulator of this pathological response. Moreover, results from *Irf8^R294C^* ^/+^ F1 heterozygotes indicate that *Irf8* regulates key pro-inflammatory cells and pathways in a gene dosage dependent fashion.

In addition to its established role in ontogeny and function of myeloid cells, *Irf8* is required for certain aspects of B lymphocyte development and T lymphocyte function including Th1 and Th17 responses [5], [23], [50]. Cellular immunophenotyping was conducted in naïve and PbA-infected tissues to identify the cell population(s) associated with and most likely responsible for pathological inflammation, whose absence is associated with ECM-resistance in BXH2 and in [BXH2×B6]F1 mice. These studies showed that a) ECM-resistance is independent of the immature myeloid hyperplasia (CD11b^+^/Ly6C^+^) characteristic of BXH2, as this trait is absent from resistant [BXH2×B6]F1 (Figure 6A), and b) is linked to brain infiltration of both myeloid (CD11b^+^/Ly6C^+^) and lymphoid cells (TCRβ^+^/CD4^+^ and TCRβ^+^/CD8^+^ T cells) in B6 mice (Figure 1E), and c) is concomitant with reduced production of IL12p40 by myeloid cells and impaired production of IFNγ by T cells following infection with *P. berghei*.

To identify the gene dosage dependent pathways that are activated by *Irf8* during neuroinflammation, we compared brain transcript profiles from PbA-infected B6 and BXH2 mice and extracted a list of genes that are induced by infection in an *Irf8*-dependent and independent fashion (two-factor ANOVA and pairwise analyses) (Figure 2, Table S1). In parallel, we carried out ChIP-seq experiments to map genome-wide IRF8 binding sites. We compared the position of these binding sites to the gene lists generated by transcript profiling and identified both IRF8-bound genes (Table S2), and IRF8-bound genes regulated in an *Irf8*-allele specific fashion (Tables S1). There was substantial overlap between these gene sets, which were similarly dominated by markers of antigen-presenting cells (APC) including antigen processing and presentation, production of type I interferon, pro-inflammatory cytokines/chemokines and others. These analyses confirm that *Irf8* plays a prominent role in the unique functions of APCs including antigen capture and microbial phagocytosis (*C1q, C4b, Fcgr4, Fcgr1*), cytoplasmic inflammasome platforms (*Nlrc5*, *Ifi205*), phagosome maturation and recruitment of small GTPases (*Irgm1, Irgm2, Igtp, Gbp2, Gbp3*), endoplasmic reticulum membrane associated antigen transport (*Tap1, Tap2*), and both Class I and Class II MHC-dependent antigen presentation in APCs (*B2m, H2-A, D, K, L, Q, T* molecules). These gene lists also featured a number of inflammatory cytokines and chemokines involved in chemotaxis of myeloid and lymphoid cell types to the sites of infection (*Ccl4, Ccl5, Ccl7, Ccl12, Cxcl9, Cxcl10*). Network analysis of the IRF8 targets bound and activated in response to PbA infection confirms that *Irf8* directly regulates several myeloid-specific, pro-inflammatory pathways that are ultimately responsible for pathological inflammation (Figure S1).

These findings are compatible with a simple functional model where myeloid cells (including APCs) and lymphoid cells are rapidly recruited in large numbers to the site of PbA infection and associated tissue injury - namely capillaries of the blood brain barrier. This initiates a robust IRF8-dependent pro-inflammatory cascade. Local amplification of this response by recruited cells leads to excessive production of immunopathological soluble mediators such as IFNγ and TNFα by T lymphocytes and induces other transcription factors including *Stat1* and other IRF family members (*Irf1, Irf7, Irf9*). Damage to the BBB causes further infiltration of pro-inflammatory cytokine-secreting cells (Figure 1E), and ultimately appearance of lethal neurological symptoms in susceptible mice. Absence of *Irf8* blunts this pathological response and allows mutant BXH2 mice to avoid developing neuroinflammation during ECM, thus surviving the critical acute phase. Results from immunophenotyping studies (Figures 6–7) indicate that the protective effect of *Irf8* (and *Irf1*) mutations is associated with simultaneously diminished pro-inflammatory responses by both the myeloid and the lymphoid compartments; this is most evident for the blunted production of IFNγ in ECM-resistant BXH2, *Irf1^−/−^* or BXH2×B6]F1 mice, when compared to ECM-susceptible B6 (serum level, and by CD4^+^ and CD8^+^ T cells with or without stimulation with PMA/ionomycin).

At steady-state, BXH2 mice show a severe depletion of myeloid DC subsets but display expansion of immature myeloid progenitors, including granulocytes-like cells (Cd11b^+^/Ly6C^+^) in peripheral tissues [12]–[15]. Literature in support of [51], [52] or arguing against [53] a role for granulocytes/neutrophils in the pathogenesis of ECM has been published. However, myelocytic hyperplasia in BXH2 is not responsible for ECM resistance, since [BXH2×B6]F1 mice are also resistant and do not display this myeloid expansion (Figures 1A and 6). Although the depletion of DCs, along with a concomitant reduction in IL-12 production and antigen-specific T-cell priming in BXH2 is likely to account for a significant component of ECM-resistance, we propose that even in the context of normal myeloid cell numbers, reduced IRF8-dependent transcriptional activation of APC-specific and T-cell specific pathways is sufficient to significantly blunt inflammatory response and protect against acute pathological inflammation. This is based on several observations. First, IRF8 behaves primarily as a transcriptional activator, not a repressor, in myeloid cells as can be seen by the enrichment of IRF8 binding sites in increased genes only (Figure 3B). Table S1 also highlights that for each gene regulated in a strain and infection specific way, *Irf8*-competent mice invariably show a higher magnitude fold change than BXH2, and the majority of these genes are increased (Group 1 and 2) rather than decreased (Group 3). Second, *Irf8^R294C^* ^/+^ F1 heterozygotes show normal numbers of myeloid cells (DCs, macrophages) and lymphoid cells, but still exhibit significant resistance to PbA induced ECM (Figures 1). Thirdly, the inactivation of several direct transcriptional targets of IRF8 (identified in our study as bound and regulated by IRF8 during PbA infection) including the phagosome-associated small GTPase *Irgm1* (Figure 5), the pro-inflammatory cytokines *Il12p40* (Figure 5 and [47]), *Cxcl9* and *Cxcl10* [54], the Cd11b receptor *Icam1* [55], [56], and the transcriptional activator and IRF8 dimerization partner *Irf1* [57] have been shown to increase resistance to ECM in mouse knockout mutants (Figure 5 and Refs [58], [59]). Finally, inactivation of additional IRF8 targets, detected herein by ChIP-seq, have previously been shown to protect against PbA-induced ECM, including *Ifng* [60], *Jak3* [45], *Cd8*, *Cd14*, *Cd40*, *Hc*, *Fcgr2*, *Lta* and *Ltbr* [61]. Together, these results highlight the role of IRF8 in regulating pro-inflammatory pathways in myeloid and lymphoid cells during ECM-associated neuroinflammation.

Although IRF8-dependent activation of pro-inflammatory pathways in myeloid cells has detrimental and pathological consequences during PbA infection, it clearly plays a protective role in other infections including pulmonary tuberculosis. Indeed, using the same analysis and stringent statistical parameters, we noted a strong overlap between the list of genes increased in brains of B6 mice in response to PbA infection and up-regulated in lungs 30 days following aerosol infection with *M. tuberculosis* (Table S2). Of the 123 genes increased in PbA-infected brains, more than half (n = 66) were also up-regulated in *M. tuberculosis*-infected lungs, and nearly three quarters (90/123) of the PbA-regulated genes harbored an IRF8 binding site. Amongst the 66 genes up-regulated during both *M. tuberculosis* infection and during ECM, a striking 80% (n = 53) display one or more IRF8 binding sites. Furthermore, inactivating mutations in several of these common genes including *Irf8* [22], *Irf1* [62], and *Irgm1* [63] cause susceptibility to pulmonary tuberculosis, while conveying some degree of protection against ECM. Mutations in additional ECM increased genes such as *Tap1* and *B2m* also cause susceptibility to tuberculosis [64], while their direct effects on ECM susceptibility have yet to be tested. We propose that this set of 53 genes represents the core *Irf8*-dependent pro-inflammatory response pathways that play key roles in protection against TB, and pathological inflammation associated with ECM. Network analysis of these direct Irf8 targets highlights central nodes specific to myeloid cells in general, and dendritic cells in particular, including pro-inflammatory cytokines, type 1 interferon response and their transcriptional regulators.

Inactivation of *Irf8* or certain of its transcriptional targets leads to complete protection against ECM. On the other hand, reduced *Irf8* function causes partial protection (in *Irf8^R294C/+^* F1 heterozygotes). Such gene-dosage dependent effects raise the possibility that even small changes in expression or activity of IRF8 may have phenotypic consequences, with increased *Irf8* expression possibly associated with enhanced and/or chronic inflammation. Results from GWAS have pointed to *IRF8* as one of the genetic factors implicated in the complex genetic architecture of several human inflammatory conditions. For example, SNPs ∼60 kb downstream of *IRF8* are associated with increased risk for both ulcerative colitis [33] and Crohn's disease (lead SNP: rs16940202) [35], [49]. In agreement with our hypothesis, the same set of SNPs exhibit a remarkably similar association pattern with *IRF8* expression levels in colon and rectum (Gros and Georges, unpublished). The findings are consistent with the notion that one or more regulatory variants increase IBD risk by enhancing intestinal *IRF8* expression. In addition, *IRF8* is a key risk factor in multiple sclerosis (MS), and its association with this disease has been validated in multiple GWAS and meta-analyses [36], [38]. In MS, disease risk is associated with an expression SNP (rs17445836) mapping 61 kb downstream of *IRF8* which is associated with both disease susceptibility and higher peripheral blood mononuclear cell *IRF8* mRNA levels [36]. Finally, a SNP near *IRF8* was found associated with systemic lupus erythematosus [65], a disease where production of type I interferon is central to pathogenesis. These results not only support a role for *IRF8* in human chronic inflammatory conditions but further suggest that, in agreement with our results in mice, even modest changes in expression or activity of *Irf8* in the context of persistent microbial or autoimmune stimulus, may lead to chronic or pathological inflammation. Furthering this proposal, we note that several IRF8 targets regulated during neuroinflammation in PbA-infected mice have also been detected as genetic risk factors in GWAS of human chronic inflammatory conditions, including the MHC (type 1 diabetes, rheumatoid arthritis, lupus, MS, psoriasis), *CCL7* (IBD), *IRF1* (IBD), *IRF7* (Lupus) and *ICAM1* (IBD) (Table S3). This highlights the role of IRF8 and its regulated pathways in pathological inflammation in humans.

The mouse model of acute neuroinflammation induced by PbA infection has proven valuable to identify novel genes, proteins and pathways involved in pathological inflammatory conditions. This model may help prioritize genes identified in human GWAS for therapeutic development, including assessing activity of novel anti-inflammatory drug candidates for use in common human inflammatory conditions.

## Materials and Methods

### Ethics statement

All mice were kept under specific pathogen free conditions and handled according to the guidelines and regulations of the Canadian Council on Animal Care. Mice experimentation protocol was approved by the McGill Facility Animal Care Committee (protocol number: 5287).

### Mice

C57BL/6J (B6), BXH2, *Il12p40^−/−^*, *Irf1^−/−^*, and *Isg15^−/−^* mutant mice were obtained from The Jackson Laboratory (Bar Harbor, ME). *Stat1^−/−^* mutant mice were purchased from Taconic Farms (Germantown, NY). *Ifng^−/−^* deficient mice were obtained from Dr. M. M. Stevenson (Montreal General Hospital Research Institute), *Ifit1^−/−^* mutant mice were obtained from Dr. M. Diamond (Washington University School of Medicine, St-Louis), *Irgm1^−/−^* from Dr. J. D. MacMicking (Yale, New Haven, CT) and *Nlrc4^−/−^* from Millenium Pharmaceuticals, Inc. and Dr. R. A. Flavell (Yale, New Haven, CT). [BXH2×B6]F2 mice were generated by inter-crossing [BXH2×B6]F1 mice.

### Parasites and infection

*P. berghei* ANKA (PbA) was obtained from the Malaria Reference and Research Reagent Resource Centre (MR4), and was stored frozen at −80°C. Prior to experimental infections, PbA was passaged in B6 mice until peripheral blood parasitemia levels reached 3–5%, at which point animals were euthanized by CO_2_ inhalation, exsanguinated and an infectious stock was prepared. All experimental infections were done via intraperitoneal (i.p.) injection of 10^6^ parasitized red blood cells (pRBC). Blood parasitemia was monitored during infection by microscopic examination of thin-blood smears stained with Diff-Quick (Dade Behring, Newark, DE, USA). The appearance of neurological symptoms (shivering, tremors, ruffled fur, seizures, paralysis) associated with cerebral malaria (ECM) was monitored closely, and affected animals were immediately sacrificed as previously described [39]. Survival curves were compared using Kaplan-Meier statistics.

### Evans Blue dye extravasation assay

To monitor the integrity of the blood brain barrier during experimental ECM, groups of control and PbA-infected B6 and BXH2 mice were injected intraperitoneally with 0.2 ml of 1% Evan's Blue dye (E2129; Sigma-Aldrich, Oakville, ON, Canada) in sterile phosphate-buffered saline (PBS) on d7 and d16 (BXH2 only) post-infection (n = 3 mice/condition). The dye was allowed to circulate for 1 h, then the mice were sacrificed by CO_2_ inhalation, perfused with PBS and the brains were dissected and photographed.

### Cytokines determination

B6, BXH2, [BXH2×B6]F1 and *Irf1^−/−^* mice were infected with PbA and sacrificed at day 6 post-infection. Whole blood was collected by cardiac puncture and serum separated by centrifugation. Levels of circulating IL12p40 and IFNγ were measured using a commercially available ELISA kit, according to manufacturer's instructions (Biolegend and eBioscience, respectively). Spleens from PbA-infected mice were collected, single-cell suspensions prepared and re-suspended in complete RPMI. 4×10^6^ cells were plated in 48-well tissue culture plates and were stimulated with PMA/Ionomycin (eBioscience) or IL12p70 (Biolegend) for 48 hours. Culture supernatants were collected and assayed for IL12p40 and IFNγ production.

### Flow cytometry analysis

Naïve or PbA infected B6, BXH2, and [BXH2×B6]F1 mice were sacrificed, exsanguinated and perfused with PBS containing 2 mM EDTA. Spleens were collected, single-cell suspensions prepared and resuspended in FACS buffer (PBS, 2% FBS, 0.02% NaN_3_). Infiltrating brain leukocytes were enriched by Percoll gradient centrifugation, as previously described [66]. Briefly, brains were gently disrupted using a dounce homogenizer, and then separated over discontinuous 70/30% Percoll gradients. Cells from the interface were collected, washed and resuspended in FACS buffer. Cells were surface stained for 30 minutes in the dark at 4°C with the following cocktails: APC-eFluor780 anti-CD45 (30-F11), APC anti-TCRβ (H57-597), PE anti-CD4 (GK1.5), FITC anti-CD8a (53-6.7) to stain lymphoid cells, or APC-eFluor780 anti-CD45 (30-F11), PE-Cy7 anti-CD11b (M1/70), PE anti-Ly6C (HK1.4), BV421 anti-Ly6G (1A8, Biolegend), eFluor660 anti-F4/80 (BM8), FITC anti-MHCII (M5/114.15.2), and PerCP-Cy5.5 anti-CD11c (N418) to stain myeloid cells. Acquisition was performed using an eight-color FACS Canto II flow cytometer (BD Biosciences) and data analyzed using FlowJo software (Tree Star). For the spleen, between 5×10^4^ and 10^5^ live cells were acquired; for the brain, a maximum number of cells was acquired, but did not exceed 5×10^3^. Cell aggregates were gated out based on the forward scatter (FSC)-height versus FSC-area plot and the live cell gate established based on the side scatter (SSC)-area versus FSC-area. Leukocytes were gated as CD45+ cells in the spleen and as CD45^hi^ cells in the brain; microglia were excluded based on CD45^low^ staining. Data were expressed as the percentage of total CD45^+^ cells or absolute numbers according to total splenocyte numbers (Figure S2B). All antibodies are from eBioscience, unless otherwise stated.

### Transcript profiling

Whole brains were dissected from B6 and BXH2 mice either prior to (d0) or d7 post infection (n = 3/condition). Total brain RNA was isolated using TRIzol reagent (Invitrogen, Burlington, Canada) according to the manufacturer's instructions, followed by further purification with RNeasy columns (Qiagen, Toronto, Canada) and hybridized to Illumina MouseWG-6 v2.0 microarrays at Genome Quebec Innovation Centre, Montreal, Canada. Unsupervised principal components analysis was done in R, using the lumi [67] package to transform with vst (variance stabilizing transformation) and to perform quartile normalization. For other analyses, microarray expression data was log2-transformed, median normalized and analyzed using GeneSifter (Geospiza) software. Groups were compared using either pairwise t-tests (≥2-fold cutoff, Benjamini-Hochberg corrected p_adj_-values<0.05) or two-factor ANOVA (≥2-fold cutoff, Benjamini-Hochberg corrected p_adj_-values<0.05) to identify genes whose expression is modulated in a strain-dependent, infection-dependent and/or interactive fashion. Lists of genes that were differentially expressed were clustered according to fold change using Multi Experiment Viewer [68]. Raw data can be accessed through the Gene Expression Omnibus (# pending).

### Chromatin immunoprecipitation (ChIP)

The J774 mouse macrophage cell line was grown to 80% confluence in complete Dulbecco's modified Eagle's medium (DMEM). The cells plated in 150 mm tissue culture-grade Petri dishes (Corning Inc., Corning, NY) were treated with 400 U/ml IFNγ (Cell science, Canton, MA) and CpG DNA oligonucleotides (5′-TCCATGACGTTCCTGACGTT-3′) for 3 h. Chromatin immunoprecipitations were performed as previously described with few modifications [69]. Briefly, treated cells were crosslinked for 10 min at 20°C with 1% formaldehyde in culture medium. Crosslink was stopped with ice-cold PBS containing 0.125 M glycine for 5 min. Nuclei were prepared and chromatin was sonicated with a Branson Digital Sonifier (Branson Ultrasonics, Danbury, CT) to an average size of 250 bp. Sonicated chromatin was incubated overnight on a rotating platform at 4°C with a mixture of 20 µl Protein A and 20 µl Protein G Dynabeads (Invitrogen, Carlsbad, CA) pre-bound with 6 µg of normal goat IgG (sc-2028) or IRF8 (sc-6058×) antibodies (Santa Cruz Biotechnologies, Santa Cruz, CA). Immune complexes were washed sequentially for 2 min at room temperature with 1 ml of the following buffers: Wash B (1% Triton X-100, 0.1% SDS, 150 mM NaCl, 2 mM EDTA, 20 mM Tris-HCl pH 8), Wash C (1% Triton X-100, 0.1% SDS, 500 mM NaCl, 2 mM EDTA, 20 mM Tris-HCl pH 8), Wash D (1% NP-40, 250 mM LiCl, 1 mM EDTA, 10 mM Tris-HCl pH 8), and TEN buffer (50 mM NaCl, 10 mM Tris-HCl pH 8, 1 mM EDTA). After decrosslinking, the DNA was purified with QIAquick PCR purification columns following manufacturer's procedure (Qiagen, Mississauga, Ca). IRF8 ChIP efficiency relative to the IgG control was assessed by qPCR using the Perfecta SYBR green PCR kit (Quanta Bioscience, Gaithersburg, MD) for known IRF8 binding sites [3] (oligonucleotide sequences are available upon requests).

### ChIP-seq preparation and analysis

A total of 8 independent ChIPs were pooled for each condition (IRF8 and IgG). Libraries and flow cells were prepared following Illumina's recommendation (Illumina, San Diego, CA), with a size selection step targeting fragments between 250 and 500 bp. The ChIP libraries were sequenced on Illumina HiSeq 2000 sequencer. The sequencing yielded 86 and 79 million 50 bp sequence reads for IgG control and IRF8 samples, respectively. The reads were mapped to the mouse mm9 genome assembly using Bowtie with the following parameters: -t –solexa1.3-qual –sam –best mm9 [70]. The mapping efficiency was 91.7% for IgG and 91.9% for IRF8 samples. To identify IRF8 binding peaks, we used the MACS 1.4.1 peak finder with the following parameters: –bw 250 –mfold 7,30 –pvalue 1e-5 -g mm [42]. This analysis yielded 11216 genomic regions bound by IRF8 with p-values under the threshold of 10^−5^. The genes identified as affected by PbA infection in the expression profiling experiment were queried for the presence IRF8 binding peaks in a 20 kb interval around the gene transcription start site (TSS). This analysis was also performed for all the genes represented on the Illumina mouse WG-6 v2.0 array used in the microarray experiments, to assess the background association of IRF8 peaks with surrounding genes (Figure 3B).

### Gene network analysis

The list of all genes differentially regulated in B6 mice during infection (B6 d7/d0 pairwise) was uploaded into Ingenuity Pathway Analysis and networks were generated based on known direct or indirect interactions from published reports and the IPA databases. Seventeen networks were constructed and the three most significant were re-drawn in Adobe Illustrator CS4 14.0.0 (Adobe Systems Inc.). IRF8 binding sites from ChIP-seq data were cross-referenced and genes were colored according to their pairwise fold change during infection for each strain (Figure S1).

A second set of networks was generated according the same procedure using the list of 53 genes detailed in Table S1 possessing at least one IRF8 binding site and up-regulated by both PbA and Mtb infection (Figure 4).

## Supporting Information

Figure S1 **Network analysis of genes regulated by PbA infection in B6 mice.** Gene interaction networks were adapted from those generated using Ingenuity Pathway Analysis and the three top scoring networks are depicted. Genes are indicated by circles with a blue ring to indicate those with IRF8 binding sites while arrows represent direct (black) or indirect (gray) biological connections within the networks. Left panels are colored according to fold change during infection in B6 mice while the right panels are colored by fold change in BXH2 mice. Multi-member gene families are indicated by a gradient rather than a single color. (A and B) Cellular signaling and hematology network, (C and D) interferon signaling and inflammatory network and (E & F) antigen presentation response. For clarity, indirect connections are not shown in panels C and D which feature highly interconnected gene sets.
(TIF)

Figure S2 **Gating strategy for immunophenotyping of spleen cells from PbA-infected mice.** The gating strategy is shown for representative specimens of B6 (A) and BXH2 (B) mice. An initial gate to isolate single cells was established based on the FSC-H and FSC-A. Cellular debris and dead cells were then excluded by SSC-A and FSC-A gating, and leukocytes selected according to the CD45^+^ staining. For the myeloid compartment, granulocytes were identified as CD11b^+^Ly6G^+^ and monocytes/macrophages as CD11b^+^F4/80^+^. Myeloid dendritic cells were first gated as Ly6C^−^CD11b^+^ (excluding granulocytes and monocytes) and then further specified as CD11c^+^MHCII^+^. CD8^+^ and CD4^+^ T cells were identified as CD8^+^CD4^−^ and CD4^+^CD8^−^ staining. Every counter plot title denotes the gate within. (C) The characteristic BXH2 immature myeloid expansion, as shown in Figure 6A, is comprised of monocyte-like (Gate R1 cells are F4/80^+^ and Ly6G^−^) and granulocyte-like cells (Gate R1 cells are F4/80^−^ and Ly6G^+^). (D) Total number of spleen cells used to report cell type numbers in Figure 6 and 7.
(TIF)

Figure S3 **Total cell counts in spleen prior and after PbA infection.** (A) Absolute number of splenocytes and leukocyte populations in B6, BXH2 and [BXH2×B6]F1 mice prior to infection (day 0). As shown in Figure 6A, BXH2 splenomegaly is associated with an immature myeloid cell hyperplasia. B6 and [BXH2×B6]F1 mice have highly similar cell numbers, with the exception of [BXH2×B6]F1 exhibiting a BXH2-like reduced number of CD8^+^ T. (B) Total cell numbers in mice 6 days after PbA infection. This collection of graphs is reproduced from Figure S2, 6 and 7 for a basis of comparison with the day 0 values shown in (A).
(TIF)

Table S1 **Strain and infection specific regulation of gene expression in PbA infected B6 and BXH2 mice.** Fold change of transcripts d7/d0 is shown, asterisks indicate that the fold change reported is the average of two or more probes (two-way ANOVA, 2>fold change cut-off, p_adj_-interaction<0.05). Gene order is as seen in Figure 3C; and bold text indicates genes with at least one IRF8 binding site within 20 kb of the transcription start site.
(PDF)

Table S2 **Genes regulated in B6 mouse brains during PbA infection, and comparison to infection-induced fold change to what has been reported in the lungs of B6 mice during** ***M. tuberculosis*** **infection.** The number of IRF8 ChIP-seq binding peaks within 20 kb of the TSS is also displayed where applicable. Genes bearing one or more IRF8 binding sites, and regulated in both cerebral malaria and pulmonary tuberculosis are highlighted in yellow.
(PDF)

Table S3 **Transcriptional response to** ***P. berghei*** **in CM-susceptible B6 mice, sorted according to ontology category (AMIGO).** IRF8 targets (indicated by bold text) are significantly enriched in upregulated genes. Superscript labels refer to genes where the human ortholog has been identified in GWAS studies for psoriasis (P), rheumatoid arthritis (RH), celiac disease (C), Crohn's disease (CD), ulcerative colitis (UC), diabetes (D), multiple sclerosis (MS), systemic lupus erythematous (SLE), irritable bowel disease (IBD), or where the human ortholog is found in the MHC (MHC).
(PDF)
