# Correction: Irf8-Regulated Genomic Responses Drive Pathological Inflammation during Cerebral Malaria

**DOI:** 10.1371/journal.ppat.1004719

**Published:** 2015-03-04

**Authors:** The PLOS Pathogens Staff

The Gene Expression Omnibus accession number is missing from the Materials and Methods section, from the last sentence of the “Transcription profiling” subsection. The corrected sentence is: Raw data can be accessed through the Gene Expression Omnibus (GSE65036).
