# Supplementary figures and images for: Irf8-Regulated Genomic Responses Drive Pathological Inflammation during Cerebral Malaria
Source: PLoS Pathog. 2013 Jul 11;9(7):e1003491. doi: 10.1371/journal.ppat.1003491 (PMC3708918; doi:10.1371/journal.ppat.1003491)

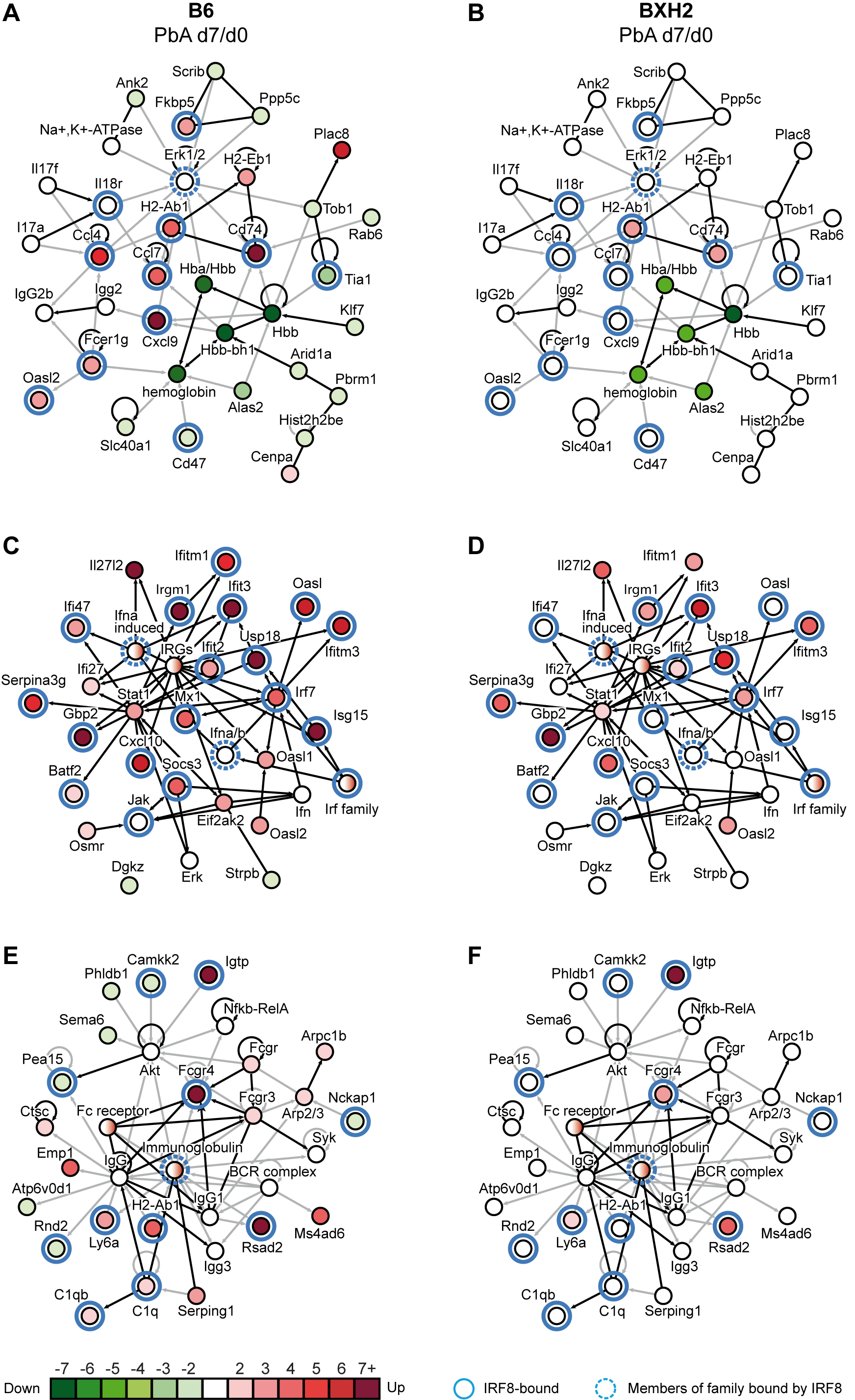

Supplement: Figure S1 — Network analysis of genes regulated by PbA infection in B6 mice. Gene interaction networks were adapted from those generated using Ingenuity Pathway Analysis and the three top scoring networks are depicted. Genes are indicated by circles with a blue ring to indicate those with IRF8 binding sites while arrows represent direct (black) or indirect (gray) biological connections within the networks. Left panels are colored according to fold change during infection in B6 mice while the right panels are colored by fold change in BXH2 mice. Multi-member gene families are indicated by a gradient rather than a single color. (A and B) Cellular signaling and hematology network, (C and D) interferon signaling and inflammatory network and (E & F) antigen presentation response. For clarity, indirect connections are not shown in panels C and D which feature highly interconnected gene sets. (TIF) [file ppat.1003491.s001.tif]

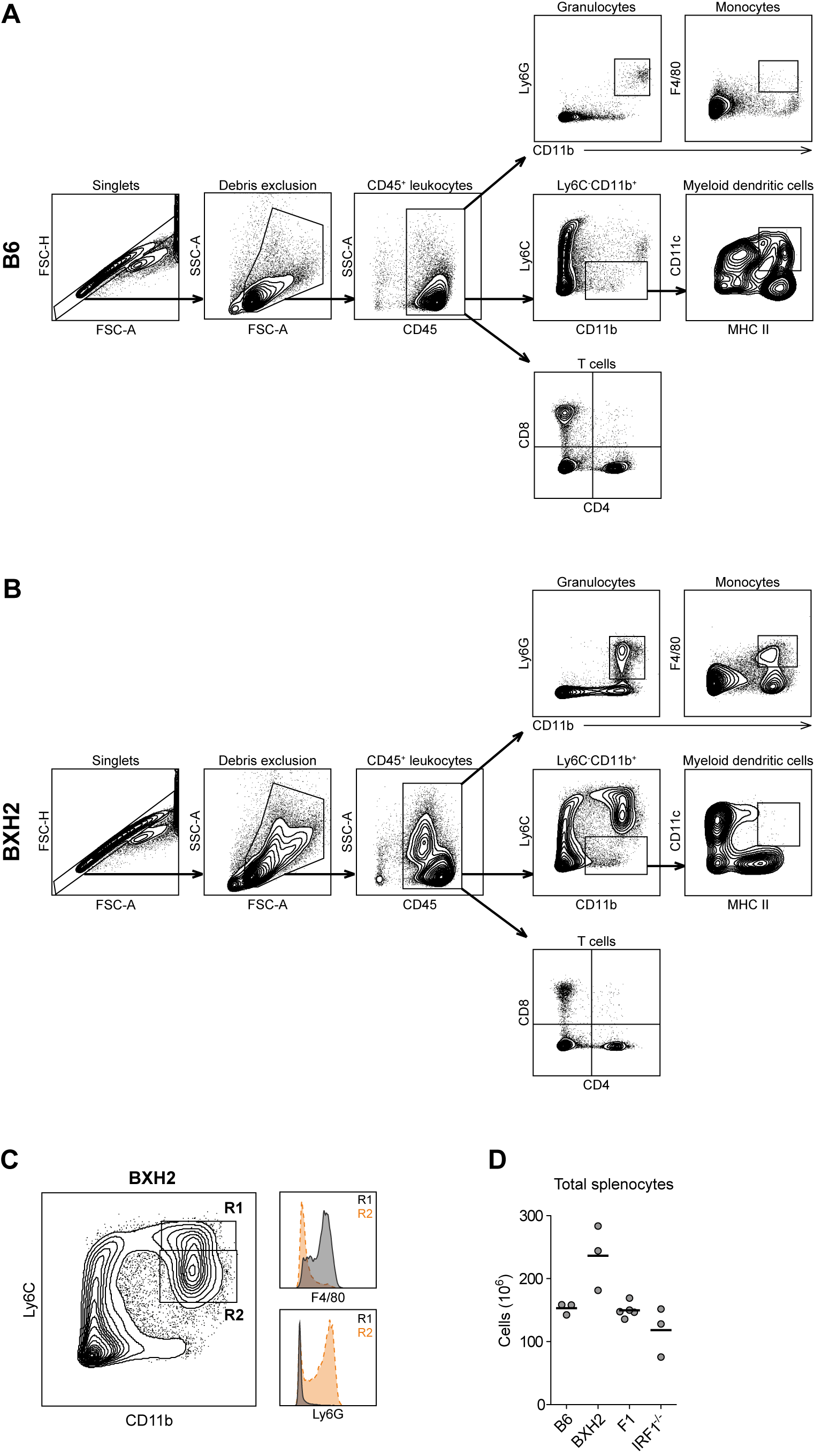

Supplement: Figure S2 — Gating strategy for immunophenotyping of spleen cells from PbA-infected mice. The gating strategy is shown for representative specimens of B6 (A) and BXH2 (B) mice. An initial gate to isolate single cells was established based on the FSC-H and FSC-A. Cellular debris and dead cells were then excluded by SSC-A and FSC-A gating, and leukocytes selected according to the CD45+ staining. For the myeloid compartment, granulocytes were identified as CD11b+Ly6G+ and monocytes/macrophages as CD11b+F4/80+. Myeloid dendritic cells were first gated as Ly6C−CD11b+ (excluding granulocytes and monocytes) and then further specified as CD11c+MHCII+. CD8+ and CD4+ T cells were identified as CD8+CD4− and CD4+CD8− staining. Every counter plot title denotes the gate within. (C) The characteristic BXH2 immature myeloid expansion, as shown in Figure 6A, is comprised of monocyte-like (Gate R1 cells are F4/80+ and Ly6G−) and granulocyte-like cells (Gate R1 cells are F4/80− and Ly6G+). (D) Total number of spleen cells used to report cell type numbers in Figure 6 and 7. (TIF) [file ppat.1003491.s002.tif]

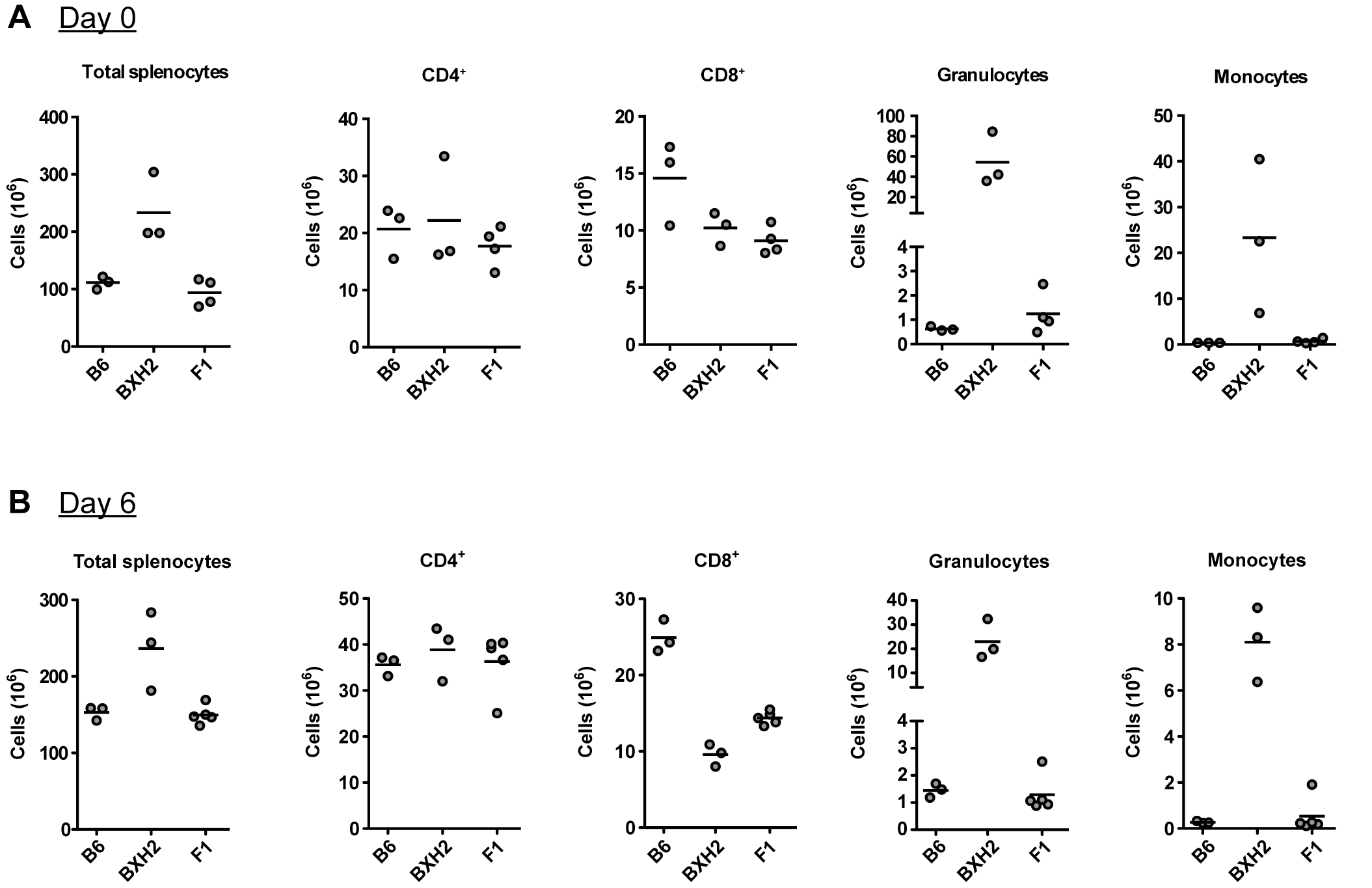

Supplement: Figure S3 — Total cell counts in spleen prior and after PbA infection. (A) Absolute number of splenocytes and leukocyte populations in B6, BXH2 and [BXH2×B6]F1 mice prior to infection (day 0). As shown in Figure 6A, BXH2 splenomegaly is associated with an immature myeloid cell hyperplasia. B6 and [BXH2×B6]F1 mice have highly similar cell numbers, with the exception of [BXH2×B6]F1 exhibiting a BXH2-like reduced number of CD8+ T. (B) Total cell numbers in mice 6 days after PbA infection. This collection of graphs is reproduced from Figure S2, 6 and 7 for a basis of comparison with the day 0 values shown in (A). (TIF) [file ppat.1003491.s003.tif]
