# Supplementary material for: Irf8-Regulated Genomic Responses Drive Pathological Inflammation during Cerebral Malaria
Source: PLoS Pathog. 2013 Jul 11;9(7):e1003491. doi: 10.1371/journal.ppat.1003491 (PMC3708918; doi:10.1371/journal.ppat.1003491)
Supplement: Table S1 — Strain and infection specific regulation of gene expression in PbA infected B6 and BXH2 mice. Fold change of transcripts d7/d0 is shown, asterisks indicate that the fold change reported is the average of two or more probes (two-way ANOVA, 2>fold change cut-off, padj-interaction<0.05). Gene order is as seen in Figure 3C; and bold text indicates genes with at least one IRF8 binding site within 20 kb of the transcription start site. (PDF) [file ppat.1003491.s004.pdf]

Table S1. Strain and infection specific regulation of gene expression in PbA infected B6 and BXH2 mice. Fold change of transcripts d7/d0 is shown, asterisks indicate that the fold change reported is the average of two or more probes (two-way ANOVA, 2>fold change cut-off, p_{adj}-interaction<0.05). Gene order is as seen in Figure 2C; and bold text indicates genes with at least one IRF8 binding site within 20kb of the transcription start site.

Group 1 genes			Group 2 genes			Group 3 genes		
**GeneID**	**B6**	**BXH2**	**GeneID**	**B6**	**BXH2**	**GeneID**	**B6**	**BXH2**
**Igtp**	**17.29**	**6.56**	**Xaf1**	**5.22**	**2.40**	H2afv	-1.44	1.06
**Cxcl10**	**14.90**	**3.18**	**Ccl12***	**5.67**	**1.62**	EG381438	-1.36	1.07
**Rsad2**	**12.79**	**3.94**	**Psmb9**	**6.09**	**1.16**	Prkag2	-1.39	-1.06
**Fcgr4**	**11.50**	**2.33**	Cdkn1a*	5.17	-1.36	Tmcc1	-1.42	1.00
**Cd274**	**10.38**	**4.03**	Serpina3k	4.71	-1.11	Atp2c1	-1.40	1.01
**Gbp3***	**9.58**	**4.03**	Fosb	4.19	-1.34	1700123O20Rik	-1.35	-1.00
**Irgm1**	**9.44**	**2.76**	**Socs3**	**4.19**	**1.14**	Ccng2	-1.33	1.00
**Isg15**	**7.61**	**3.99**	**Ccl5**	**4.16**	**1.24**	**Arl8b**	**-1.25**	**1.01**
**Oasl2**	**8.31**	**3.25**	Ms4a6d	4.07	1.21	Slc4a4	-1.39	-1.14
Plac8	7.41	2.49	Emp1	3.81	1.10	Emid2	-1.57	-1.15
**Cd74***	**6.66**	**2.97**	**Ccl7***	**3.77**	**1.15**	Hes5	-2.68	-1.54
**Irf1***	**6.29**	**2.24**	**C4b***	**4.54**	**1.32**	Gga3	-2.24	-1.41
**Cxcl9**	**8.25**	**1.57**	**H2-Ab1***	**4.66**	**1.37**	D14Abb1e	-2.41	-1.29
**Ccl4***	**7.20**	**1.39**	**Tap1**	**4.63**	**1.63**	Flt1	-2.89	-1.29
Plin4	7.11	-2.20	**Cd52**	**4.19**	**1.57**	Hcn3	-2.01	-1.31
			**Chi3l4**	**4.15**	**1.71**	Akap1	-2.14	-1.25
			**Tap2**	**3.39**	**1.40**	Zbtb44	-2.05	-1.26
			**Lyz1**	**3.62**	**1.36**	**Zfp523**	**-2.07**	**-1.21**
			**Nlrc5**	**3.61**	**1.94**	E330009J07Rik	-2.45	-1.14
			Fkbp5	3.07	-1.44	Pak1	-2.11	-1.12
			Map3k6*	2.85	-1.13	**Ccm2**	**-2.07**	**-1.12**
			Atp5k	2.72	-1.09	E2f6	-2.00	-1.08
			**Angptl4**	**2.71**	**-1.08**	6330407J23Rik	-2.04	-1.05
			**Txnip***	**2.60**	**-1.09**	Fcrls	-2.15	-1.03
			AA467197	3.37	1.15	Slco1c1	-2.27	-1.03
			Slc10a6	3.23	1.01	2510022D24Rik	-1.90	1.01
			Mt2	3.31	-1.08	Raf1	-1.67	1.02
			H2-L	2.89	1.08	Mettl17	-2.27	1.08
			Sult1a1	3.01	1.03	Slc38a5	-2.95	1.12
			**Slc15a3**	**3.04**	**1.15**	**Tia1**	**-3.07**	**1.03**
			Ch25h	3.00	1.22			
			Serping1*	2.91	1.24			
			Tagln2	2.81	1.28			
			**Fpr2**	**2.80**	**1.43**			
			Ifi47	3.01	1.60			
			Oasl1	3.01	1.46			
			**Samhd1**	**3.10**	**1.50**			
			**Icam1**	**3.10**	**1.36**			
			**Psmb8**	**2.40**	**1.64**			
			**Batf2**	**2.26**	**1.50**			
			Osmr	2.39	1.23			
			**Ifi205**	**2.47**	**1.34**			
			**Ubd**	**2.63**	**1.18**			
			**Cyba**	**2.29**	**1.12**			
			Saa3	2.26	1.07			
			**Bcl2a1b**	**2.35**	**1.05**			
			H2-Eb1	2.41	1.05			
			**Bcl2a1d**	**2.48**	**1.05**			
			2410039M03Rik	2.41	-1.09			
			**Mobp**	**2.37**	**-1.10**			

	Pnpla2	2.28	-1.13		
	Gna13	2.28	-1.06		
	Cenpa	2.20	-1.32		
	Synpo	2.21	-1.16		
	Itgad*	2.03	-1.07		
	**Ugt1a6a**	**2.05**	**-1.02**		
	Adamts9	2.12	1.02		
	Phyhd1	2.19	-1.03		
	Fcgr3	2.14	1.09		
	**Ctsc**	**2.07**	**1.10**		
	**Upp1***	**2.11**	**1.14**		
	Arpc1b	2.04	1.05		
