# Supplementary material for: Irf8-Regulated Genomic Responses Drive Pathological Inflammation during Cerebral Malaria
Source: PLoS Pathog. 2013 Jul 11;9(7):e1003491. doi: 10.1371/journal.ppat.1003491 (PMC3708918; doi:10.1371/journal.ppat.1003491)
Supplement: Table S2 — Genes regulated in B6 mouse brains during PbA infection, and comparison to infection-induced fold change to what has been reported in the lungs of B6 mice during M. tuberculosis infection. The number of IRF8 ChIP-seq binding peaks within 20 kb of the TSS is also displayed where applicable. Genes bearing one or more IRF8 binding sites, and regulated in both cerebral malaria and pulmonary tuberculosis are highlighted in yellow. (PDF) [file ppat.1003491.s005.pdf]

Table S2. Overlap between transcripts regulated during cerebral malaria and during pulmonary tubercuosis in B6 mice. Transcript profiling identifies [a]genes whose expression is modulated >2-fold in B6 brains following infection with PbA (d7/d0), [b]genes whose expression is modulated >2-fold in B6 lungs following aerosol infection with M. tuberculosis[b], [c]Bonferroni-Hochberg corrected p-value for PbA d7 vs d0 t-test, [d]number of binding peaks defined by ChIP-seq within 20kb of the gene TSS. Yellow highlighting indicates genes who are upregulated by both infections and have at least 1 IRF8 binding peak near the TSS.

Gene ID	PbA d7/d0[a]	Mtb d30/d0[b]	adj. p-value[c]	#IRF8 peaks[d]	Gene Name
Gh	84.82		1.1E-02	1	Growth hormone
Gbp2	25.38	16.53	2.0E-03	1	Guanylate binding protein 2
Prl	22.77		1.6E-02		Prolactin
Igtp	17.29	14.99	2.3E-03	3	Interferon gamma induced GTPase
Cxcl10	14.90	92.96	1.1E-02	3	Chemokine (C-X-C motif) ligand 10
Ifit3	14.88	5.28	2.4E-03	3	Interferon-induced protein with tetratricopeptide repeats 3
Rsad2	12.79	3.93	5.3E-03	4	Viperin
Fcgr4	11.50	13.19	3.1E-03	2	Fc receptor, IgG, low affinity IV
Cd274	10.38	11.25	3.1E-03	2	CD274 antigen
Irgm1	9.44	10.82	5.3E-03	1	Immunity-related GTPase family M member 1
Gbp3	9.40	12.61	2.0E-03	2	Guanylate binding protein 3
Cxcl9	8.25	413.81	8.8E-03	1	Chemokine (C-X-C motif) ligand 9
Ifi27l2a	7.91	2.22	5.3E-03		Interferon stimulated gene 12
Usp18	7.68	3.63	5.3E-03	3	Ubiquitin specific protease 18
Isg15	7.61		2.0E-03	1	ISG15 ubiquitin-like modifie
Plac8	7.41	4.20	7.6E-03		C15 protein
S3-12	7.11	-4.02	1.9E-02		Perilipin
Cd74	6.51	2.25	2.5E-03	2	CD74 antigen
Irf1	6.49	3.53	2.5E-03	2	Interferon regulatory factor 1
Rnf213	6.38	2.06	2.4E-03	2	D11Ertd759e
Lgals3bp	6.36	3.86	6.5E-03		Lectin, galactoside-binding, soluble, 3 binding protein
Psmb9	6.09	8.46	2.0E-03	5	Proteasome (prosome, macropain) subunit, beta type 9 (large multifunctional peptidase 2)
Ccl12	6.06	10.68	1.0E-02	1	Chemokine (C-C motif) ligand 12
Irgm2	5.79	7.88	2.7E-02	2	Immunity-related GTPase family M member 2
Serpina3f	5.63		5.3E-03	1	serine (or cysteine) peptidase inhibitor, clade A, member 3F
Oasl2	5.58	5.97	8.7E-03	1	2-5 oligoadenylate synthetase-like 2
Ifitm3	5.57		5.0E-03	5	Interferon induced transmembrane protein 3
Serpina3g	5.49	24.45	2.5E-03	1	serine (or cysteine) peptidase inhibitor, clade A, member 3G
Xaf1	5.22		5.3E-03	1	gene model 881
Cdkn1a	5.17		4.5E-02		Cyclin-dependent kinase inhibitor 1A
Ifitm1	4.84		1.2E-02	1	Interferon induced transmembrane protein 1
Parp14	4.74	2.78	8.8E-03	2	Poly (ADP-ribose) polymerase family, member 14
Ccl4	4.71	4.51	2.7E-02	4	Strain SJL/J small inducible cytokine A4
C4b	4.64		8.8E-03	1	Complement component 4B
Tap1	4.63		6.9E-03	5	Transporter 1, ATP-binding cassette, sub-family B
Cd52	4.19	7.79	2.3E-02	3	CD52 antigen
Socs3	4.19	3.06	4.5E-02	2	Suppressor of cytokine signaling 3
H2-K1	4.17	7.92	3.0E-03	**1**	MRNA similar to histocompatibility 2, D region locus 1
Ccl5	4.16	23.91	2.4E-02	4	Chemokine (C-C motif) ligand 5
Chi3l4	4.15		5.6E-03	1	Chitinase 3-like 4
Ms4a6d	4.07	18.49	1.5E-02		Membrane-spanning 4-domains, subfamily A, member 6D
Emp1	3.81		3.2E-02		Epithelial membrane protein 1

H2-Ab1	3.08	7.76	5.3E-03	1	histocompatibility 2, class II antigen A, beta 1
Mx2	3.75		1.9E-02	1	Myxovirus (influenza virus) resistance 2
H2-D1	3.19	4.07	5.9E-03	4	MHC class Ib antigen Qa-1
Irf7	3.65	8.19	2.0E-03	1	Interferon regulatory factor 7
Lyz1	3.62		1.0E-02	2	Lysozyme 1
Nlrc5	3.61	8.55	2.0E-03	1	expressed sequence AI451557
Tap2	3.39	4.21	5.6E-03	4	Transporter 2, ATP-binding cassette, sub-family B
Txnip	3.36		4.1E-02	2	Thioredoxin interacting protein
Chi3l3	3.36		2.6E-03	1	Chitinase 3-like 3
Eif2ak2	3.32	2.53	4.6E-03		Eukaryotic translation initiation factor 2-alpha kinase 2
Mt2	3.31		4.5E-02		Metallothionein 2
Samdh9l	3.30		1.0E-02	1	sterile alpha motif domain containing 9-like
Stat1	3.25	17.30	2.1E-02		Signal transducer and activator of transcription 1
Oas1g	3.22	5.59	2.5E-03	1	2-5 oligoadenylate synthetase 1G
Ifit2	3.19	8.05	6.3E-03	1	Interferon-induced protein with tetratricopeptide repeats 2
Glipr2	3.13	5.28	3.5E-02	2	GLI pathogenesis-related 2
Tgm2	3.11		3.0E-02	2	Transglutaminase 2, C polypeptide
Icam1	3.10		8.8E-03	2	Intercellular adhesion molecule 1
Psmb8	3.10	7.99	6.2E-03	5	Proteasome (prosome, macropain) subunit, beta type 8 (large multifunctional peptidase 7)
Samhd1	3.10	3.93	5.9E-03	3	SAM domain and HD domain, 1
Fkbp5	3.07		3.0E-02	**1**	FK506 binding protein 5
B2m	3.04		5.3E-03	3	Beta-2 microglobulin
Slc15a3	3.04	6.14	2.5E-02	1	Solute carrier family 15, member 3
Ifi47	3.01	8.99	6.2E-03	3	Interferon gamma inducible protein 47
Sult1a1	3.01		1.0E-02		Sulfotransferase family 1A, phenol-preferring, member 1
Ch25h	3.00	5.86	1.2E-02		Cholesterol 25-hydroxylase
8430408G22Rik	2.91		3.5E-02		RIKEN cDNA 8430408G22 gene
Ccl7	2.91	5.76	2.6E-02	1	Chemokine (C-C motif) ligand 7
Trim21	2.90	2.87	2.5E-03	3	Tripartite motif-containing 21
Serping1	2.88		1.9E-02		Serine (or cysteine) peptidase inhibitor, clade G, member 1
H2-K2	2.86		9.0E-03	1	LOC56628
H2-T22	2.85	4.82	7.6E-03	3	Histocompatibility 2, T region locus 10
Map3k6	2.85		3.3E-02		Mitogen-activated protein kinase kinase kinase 6
D14Ertd668e	2.83		6.0E-03	1	DNA segment, Chr 14, ERATO Doi 668, expressed,
-	2.81		1.6E-02		RIKEN cDNA 1200016E24 gene
Tagln2	2.81		2.0E-02		Transgelin
Fpr2	2.80	10.17	2.1E-02	2	Formyl peptide receptor 2
Irf9	2.80	2.15	1.3E-02	1	Interferon regulatory factor 9
Pglyrp1	2.80	3.50	1.8E-02		Peptidoglycan recognition protein 1
H2-Q2	2.78		1.4E-02	2	Histocompatibility 2, Q region locus 2
Angptl4	2.71		2.9E-02	1	Angiopoietin-like 4
Fcer1g	2.71	5.57	2.1E-02	1	Fc receptor, IgE, high affinity I, gamma polypeptide
Ly6a	2.68		7.5E-03	**1**	Lymphocyte antigen 6 complex, locus A
Upp1	2.65	4.24	2.2E-02	1	Uridine phosphorylase 1
Ube1l	2.64	2.60	1.1E-02	1	Ubiquitin-activating enzyme E1-like
Oasl1	2.63	3.93	2.2E-02		Oligoadenylate synthetase-like protein-2
Ubd	2.63	259.08	5.3E-03	1	Ubiquitin D
H2-L	2.59		2.7E-02	**1**	H2-L
Tspo	2.54		1.6E-02	1	Translocator protein
H2-Q7	2.52		8.8E-03	2	Histocompatibility 2, Q region locus 7
Trim25	2.51	2.40	2.2E-02	1	tripartite motif-containing 25

Anxa2	2.49		3.3E-02	1	Annexin A2
Bcl2a1d	2.48		5.0E-03	1	B-cell leukemia/lymphoma 2 related protein A1d
Ifi205	2.47	8.65	2.5E-03	1	Interferon activated gene 205
2410039M03Rik	2.41		4.6E-02		2410039M03Rik
H2-Eb1	2.41	4.78	2.5E-03		Histocompatibility 2, class II antigen E beta
Osmr	2.39		2.7E-02		Oncostatin M receptor
Mobp	2.37		4.0E-02	1	Myelin-associated oligodendrocytic basic protein
Bcl2a1b	2.35	2.63	1.3E-02	1	B-cell leukemia/lymphoma 2 related protein A1a
Bst2	2.33		2.0E-03	1	Bone marrow stromal cell antigen 2
Cyba	2.29	4.38	3.5E-02	1	Cytochrome b-245, alpha polypeptide
Pnpla2	2.28		2.9E-02		Patatin-like phospholipase domain containing 2
Batf2	2.26		4.6E-03	1	Basic leucine zipper transcription factor, ATF-like 2
Saa3	2.26	155.32	4.1E-02		Serum amyloid A 3
Cmpk2	2.24	3.44	3.1E-03	3	Cytidine monophosphate (UMP-CMP) kinase 2, mitochondrial
Itgad	2.23		4.1E-02		integrin, alpha D
Cenpa	2.20	4.28	1.1E-02		Centromere protein A
Phyhd1	2.19		4.0E-02		Phytanoyl-CoA dioxygenase domain containing 1
H2-T17	2.18		1.4E-02	3	H2-T17
Gm12250	2.16		3.0E-02	2	LOC215405
Xdh	2.16		1.3E-02	4	Xanthine dehydrogenase
Lgals9	2.15		2.4E-03		Lectin, galactose binding, soluble 9
Fcgr3	2.14	7.42	5.4E-03		Fc gamma receptor III
Ier3	2.14		4.8E-02	2	Immediate early response 3
Adamts9	2.12		2.2E-02		Adamts9
Ifi27l1	2.10		6.2E-03		Interferon, alpha-inducible protein 27 like 1
Ctsc	2.07	5.59	8.8E-03	1	Cathepsin C
Gvin1	2.06		1.6E-02	1	GTPase, very large interferon inducible 1
Ugt1a6a	2.05		7.6E-03	2	UDP glucuronosyltransferase 1 family, polypeptide A6B
Arpc1b	2.04		3.5E-02		Actin related protein 2/3 complex, subunit 1B
C1qb	2.02	13.98	2.5E-02	2	Complement component 1, q subcomponent, beta polypeptide
Atxn7l3b	-2.00		2.3E-02		predicted gene, ENSMUSG00000074747
E2f6	-2.00		3.4E-02		E2F transcription factor 6
Nckap1	-2.00		6.2E-03	1	NCK-associated protein 1
Pcyt2	-2.00		2.5E-02		Phosphate cytidylyltransferase 2, ethanolamine
Tob1	-2.00		3.1E-02		Transducer of ErbB-2.1
Abcf2	-2.01		4.6E-02	1	ATP-binding cassette, sub-family F (GCN20), member 2
Hcn3	-2.01		7.4E-03		Hyperpolarization-activated, cyclic nucleotide-gated K+ 3
Mus81	-2.01		1.8E-02		MUS81 endonuclease homolog (yeast)
Ncoa1	-2.01		1.6E-02		Nuclear receptor coactivator 1
Ntrk3	-2.01		3.0E-02		Neurotrophic tyrosine kinase, receptor, type 3
Palm	-2.01		1.3E-02		Paralemmin
Pbrm1	-2.01		2.5E-02		polybromo 1
Strbp	-2.01	-2.17	3.3E-02		RIKEN cDNA 6430510M02 gene
Arl8b	-2.02	2.23	1.1E-02	1	ADP-ribosylation factor-like 8B
D17Wsu92e	-2.02		1.4E-02	1	DNA segment, Chr 17, Wayne State University 92, expressed
Gria2	-2.02		1.5E-02		Glutamate receptor, ionotropic, AMPA2 (alpha 2)
Lonrf2	-2.02		2.9E-02		LON peptidase N-terminal domain and ring finger 2
Pltp	-2.02	-2.51	3.0E-02	2	Phospholipid transfer protein
Myt1l	-2.03		6.1E-03		Myelin transcription factor 1-like
Oxct1	-2.03		8.7E-03	1	Scot mRNA for succinyl CoA transferase

Taok1	-2.03		2.8E-02		TAO kinase 1
Zfp385b	-2.03		4.6E-03		Zinc finger protein 385B
6330407J23Rik	-2.04		2.9E-03		RIKEN cDNA 6330407J23 gene
Apba2	-2.04		2.5E-03		X11 protein mRNA, 3 end
Fam126b	-2.04		1.1E-02	1	Family with sequence similarity 126, member B
Sdr39u1	-2.04		1.6E-02	1	Short chain dehydrogenase/reductase family 39U, member 1
1190002N15Rik	-2.05		2.1E-02		RIKEN cDNA 1190002N15 gene
Atxn7l3	-2.05		2.3E-02		ataxin 7-like 3
Cyp46a1	-2.05		2.0E-02		Cytochrome P450, family 46, subfamily a, polypeptide 1
Gna11	-2.05		1.5E-02		Guanine nucleotide binding protein, alpha 11
Ppp5c	-2.05		1.9E-02		Protein phosphatase 5, catalytic subunit
Ptn	-2.05		1.7E-02		Pleiotrophin
Sept3	-2.05		2.6E-02		Septin 3
Thra	-2.05	-2.12	8.8E-03		Thyroid hormone alpha
Zbtb44	-2.05		6.2E-03		BC038156
Hist1h2bf	-2.06		1.9E-02		Histone cluster 1
Ppp1r35	-2.06		3.0E-02		RIKEN cDNA 2010007H12 gene
Ccm2	-2.07		1.6E-02	2	Cerebral cavernous malformation 2 homolog (human)
Glg1	-2.07		1.3E-02		Golgi apparatus protein 1
Tmco3	-2.07		6.3E-03	1	Transmembrane and coiled-coil domains 3
Zfp523	-2.07		8.7E-03	1	Zinc finger protein 523
-	-2.08		3.0E-02		LOC385086
Carm1	-2.08		1.3E-02		Coactivator-associated arginine methyltransferase 1
Gtrgeo22	-2.08		3.5E-02	1	Gene trap ROSA b-geo 22
Pacsin1	-2.08		3.8E-03		Protein kinase C and casein kinase substrate in neurons 1
Pde6d	-2.08		7.6E-03		Phosphodiesterase 6D, cGMP-specific, rod, delta
Rnf167	-2.08		4.0E-02	1	Ring finger protein 167
Tmem63b	-2.08		1.2E-02		Transmembrane protein 63b
Nfix	-2.09	-2.02	1.3E-02		Nuclear factor I/X
Caln1	-2.09		3.1E-02		Calneuron 1
Dgkb	-2.09		8.7E-03		Diacylglycerol kinase, beta
Mbtps1	-2.09		2.9E-02		Membrane-bound transcription factor peptidase, site 1
Rora	-2.09		3.0E-02		RAR-related orphan receptor alpha
Scrib	-2.09		1.7E-02		Scribbled homolog (Drosophila)
Shank3	-2.09	-3.49	3.8E-03		SH3/ankyrin domain gene 3
Bcl11b	-2.10	5.31	8.8E-03		B-cell leukemia/lymphoma 11B
Cops7a	-2.10		1.3E-02		COP9 (constitutive photomorphogenic) homolog, subunit 7a (Arabidopsis thaliana)
Fam178a	-2.10		1.4E-02		family with sequence similarity 178, member A
Foxq1	-2.10		8.8E-03		Forkhead box Q1
Rhobtb2	-2.10		1.6E-02	1	Rho-related BTB domain containing 2
Zfp612	-2.10		8.8E-03		Zinc finger protein 612
Eif5a	-2.11		1.6E-02	1	Eukaryotic translation initiation factor 5A
Pak1	-2.11	3.15	5.9E-03		P21 (CDKN1A)-activated kinase 1
Tspan3	-2.11		3.6E-02		Tetraspanin 3
1110012J17Rik	-2.12	-2.39	9.3E-03		RIKEN cDNA 1110012J17 gene
Dgkz	-2.12		1.8E-02		Diacylglycerol kinase zeta
Gnao1	-2.12		2.8E-02		Guanine nucleotide binding protein, alpha O
Hsd3b2	-2.12		2.7E-02		Hydroxy-delta-5-steroid dehydrogenase, 3 beta- and steroid delta-isomerase 2
Ntm	-2.13		1.5E-02		Neurotrimin
Slc38a9	-2.13		2.3E-02	1	Solute carrier family 38, member 9

Cd47	-2.14		1.6E-02	1	CD47 antigen (Rh-related antigen, integrin-associated signal transducer)
Gats	-2.14		2.2E-02		Opposite strand transcription unit to Stag3
Rab5b	-2.14		1.3E-02		RAB5B, member RAS oncogene family
Fam63b	-2.15		6.5E-03	1	MKIAA1164 protein
Fcrls	-2.15	-2.29	7.4E-03		IFGP2
Pea15a	-2.15		3.9E-02	1	Phosphoprotein enriched in astrocytes 15A
Skiv2l	-2.15		2.9E-02		Superkiller viralicidic activity 2-like (S. cerevisiae)
Usf2	-2.15		4.0E-02	3	Upstream transcription factor 2
Jph4	-2.16		1.6E-02		Junctophilin 4
Klhdc1	-2.16		7.6E-03		Kelch domain containing 1
Ltbp4	-2.16	-4.99	1.5E-02		Latent transforming growth factor beta binding protein 4 long splice variant
Zbtb7a	-2.16		2.5E-03		Zinc finger and BTB domain containing 7a
Akap1	-2.17		3.6E-02	1	A kinase (PRKA) anchor protein 1 (Akap1), nuclear gene encoding mitochondrial protein
Gtf3a	-2.17		7.6E-03		General transcription factor III A
Mau2	-2.17		1.6E-02		RIKEN cDNA 9130404D08 gene
Rasgef1a	-2.17	-2.06	6.6E-03		RasGEF domain family, member 1A
Slc40a1	-2.17		1.6E-02		Solute carrier family 40 (iron-regulated transporter), member 1
Klf7	-2.18		2.3E-02		Kruppel-like factor 7
Camkk2	-2.18		2.6E-03	1	Calcium/calmodulin-dependent protein kinase kinase 2, beta
Fam171b	-2.18		1.1E-02		Family with sequence similarity 171, member B
Kalrn	-2.18		6.3E-03		2210407G14Rik
Scamp3	-2.18		7.6E-03		CDC-like kinase 2
Mzt1	-2.19		3.0E-02		RIKEN cDNA 2410129H14 gene
Phldb1	-2.19		2.5E-03		Pleckstrin homology-like domain, family B, member 1
Sgtb	-2.19		2.0E-02		Small glutamine-rich tetratricopeptide repeat (TPR)-containing, beta
Rnd2	-2.20		2.7E-02	1	Rho family GTPase 2
Tcf4	-2.20	-2.12	7.6E-03		Transcription factor 4
Celsr2	-2.21		2.0E-02		Cadherin, EGF LAG seven-pass G-type receptor 2 (flamingo homolog, Drosophila)
Ptprd	-2.21	-2.54	1.4E-02		protein tyrosine phosphatase, receptor type, D
3110047P20Rik	-2.22		2.2E-02		RIKEN cDNA 3110047P20 gene
Dlgap1	-2.22		8.8E-03		Discs, large (Drosophila) homolog-associated protein 1
Mgat4b	-2.22		1.6E-02	3	Mannoside acetylglucosaminyltransferase 4, isoenzyme B
Usp11	-2.22		3.1E-02		Ubiquitin specific peptidase 11
Fbxo41	-2.23		9.8E-03		F-box protein 41
B4galt3	-2.24		1.6E-02	1	UDP-Gal:betaGlcNAc beta 1,4-galactosyltransferase, polypeptide 3
Fjx1	-2.24		3.4E-02		Four jointed box 1 (Drosophila)
Gga3	-2.24		6.1E-03		Golgi associated, gamma adaptin ear containing, ARF binding protein 3
Sema6d	-2.24	-2.23	1.4E-02		Sema domain, transmembrane domain (TM), and cytoplasmic domain, (semaphorin) 6D
Smpd1	-2.24		2.9E-02		Sphingomyelin phosphodiesterase 1, acid lysosomal
Rbfox1	-2.25		1.3E-02	1	Hexaribonucleotide binding protein 1
Arid1a	-2.25		7.6E-03		AT rich interactive domain 1A (SWI-like)
Bcan	-2.25		4.6E-03		Brevican
Tmod1	-2.25	-2.04	3.1E-02		Tropomodulin 1
Rab14	-2.26		1.0E-02	2	RAB14, member RAS oncogene family

Brunol4	-2.27		1.1E-02		CUGBP, Elav-like family member 4
Mettl17	-2.27		1.3E-02		D14Ertd209e
Slco1c1	-2.27		2.0E-02		Solute carrier organic anion transporter family
Tbc1d17	-2.27		5.3E-03		TBC1 domain family, member 17
Acot7	-2.28		3.7E-02	1	BACH mRNA for acyl-CoA hydrolase
Efna5	-2.28		1.5E-02		Ephrin A5
Ank2	-2.29		2.3E-02		Ankyrin 2, brain
C1qtnf4	-2.29		5.3E-03		C1q and tumor necrosis factor related protein 4,
Orf61	-2.29		9.0E-03		open reading frame 61
AI593442	-2.30		1.5E-02		expressed sequence AI593442
Jhdm1d	-2.30	2.03	2.2E-02	1	jumonji C domain-containing histone demethylase 1 homolog D (S. cerevisiae)
Spnb4	-2.30		2.0E-02	2	BetaIV-spectrin sigma1
Acvr2b	-2.32		6.3E-03		Activin receptor IIB
Elavl3	-2.39		5.3E-03		RNA-binding protein mHuC-S
Rab6	-2.34		1.2E-02		RAB6, member RAS oncogene family
Pak3	-2.35		1.1E-02		P21-activated kinase 3
Dusp8	-2.38		1.9E-02		Dual specificity phosphatase 8
Tnrc6a	-2.38		1.1E-02		Trinucleotide repeat containing 6a
Arl2bp	-2.39		5.6E-03		ADP-ribosylation factor-like 2 binding protein (Arl2bp), transcript variant 1
Mtss1l	-2.39		2.9E-02	1	Metastasis suppressor 1-like
Cacng3	-2.40		1.2E-02	1	Calcium channel, voltage-dependent, gamma subunit 3
Ppp1ca	-2.40		2.3E-02		Protein phosphatase 1, catalytic subunit, alpha isoform
Rtn1	-2.40		8.8E-03		Reticulon 1
Gabrb1	-2.41		1.6E-02		Gamma-aminobutyric acid (GABA-A) receptor, subunit beta 1
D14Abb1e	-2.41		9.7E-03		D14Abb1e
Rgs7bp	-2.42		1.5E-02		Regulator of G-protein signalling 7 binding protein
Syt4	-2.42		6.6E-03		Synaptotagmin IV
Ugt8a	-2.44		5.0E-02		UDP galactosyltransferase 8A
E330009J07Rik	-2.45		3.6E-02		RIKEN cDNA E330009J07 gene
Gjc2	-2.45		2.5E-03	1	Gap junction protein, gamma 2
Kif3a	-2.46		7.6E-03		Kinesin family member 3A
Nfib	-2.48	-5.75	6.2E-03		Strain C57BL/6J nuclear factor I/B (Nfib)
Unc13c	-2.48		3.5E-03		LOC235480
Atp6v0d1	-2.49		2.7E-02		ATPase, H+ transporting, lysosomal V0 subunit D1
Prrt1	-2.50		1.7E-02	1	Proline-rich transmembrane protein 1
4930402H24Rik	-2.50	-2.44	1.6E-02		RIKEN cDNA 4930402H24 gene
Hes5	-2.53		1.1E-02		Hairy and enhancer of split 5 (Drosophila)
Mark2	-2.59		1.3E-02		MAP/microtubule affinity-regulating kinase 2 (Mark2), transcript variant 1
Msl1	-2.59		8.8E-03		Male-specific lethal 1 homolog (Drosophila)
Gdi1	-2.60		3.1E-02		Guanosine diphosphate (GDP) dissociation inhibitor 1
Slc38a2	-2.64		2.1E-02	1	Solute carrier family 38, member 2
Psd2	-2.68		1.3E-02		Pleckstrin and Sec7 domain containing 2
Ppap2b	-2.69	-2.65	1.5E-02	1	Phosphatidic acid phosphatase type 2B
Grm4	-2.75		2.9E-02		glutamate receptor, metabotropic 4
Epn2	-2.79	-2.06	2.1E-02		Epsin 2
2210018M11Rik	-2.83		1.0E-02		2210018M11Rik
Lphn1	-2.85	-2.17	1.6E-02		latrophilin 1
Flt1	-2.89	-2.08	5.6E-03		FMS-like tyrosine kinase 1
Slc38a5	-2.95	-3.45	2.3E-02		Solute carrier family 38, member 5

Alas2	-3.04	-2.56	2.9E-02		Aminolevulinic acid synthase 2
Itm2a	-3.05		5.9E-03		Integral membrane protein 2A
Tia1	-3.07		1.5E-02	1	cytotoxic granule-associated RNA binding protein 1
Prkcb	-3.21	2.70	2.0E-02	1	Protein kinase C, beta
Cxcl12	-4.18	2.58	1.4E-02		Chemokine (C-X-C motif) ligand 12
Hbb-b1	-23.47		2.1E-02		Hemoglobin, beta adult minor chain
Hba-a1	-24.73		2.9E-02		Hemoglobin alpha, adult chain 1
