# Supplementary material for: Irf8-Regulated Genomic Responses Drive Pathological Inflammation during Cerebral Malaria
Source: PLoS Pathog. 2013 Jul 11;9(7):e1003491. doi: 10.1371/journal.ppat.1003491 (PMC3708918; doi:10.1371/journal.ppat.1003491)
Supplement: Table S3 — Transcriptional response to P. berghei in CM-susceptible B6 mice, sorted according to ontology category (AMIGO). IRF8 targets (indicated by bold text) are significantly enriched in upregulated genes. Superscript labels refer to genes where the human ortholog has been identified in GWAS studies for psoriasis (P), rheumatoid arthritis (RH), celiac disease (C), Crohn's disease (CD), ulcerative colitis (UC), diabetes (D), multiple sclerosis (MS), systemic lupus erythematous (SLE), irritable bowel disease (IBD), or where the human ortholog is found in the MHC (MHC). (PDF) [file ppat.1003491.s006.pdf]

Table S3. Transcriptional response to *P. berghei* in CM-susceptible B6 mice, sorted according to ontology category (AMIGO). IRF8 targets (indicated by bold text) are significantly enriched in upregulated genes. Superscript labels refer to genes where the human ortholog has been identified in GWAS studies for psoriasis (P), rheumatoid arthritis (RH), celiac disease (C), Crohn's disease (CD), ulcerative colitis (UC), diabetes (D), multiple sclerosis (MS), systemic lupus erythmatous (SLE), irritable bowel disease (IBD), or where the human ortholog is found in the MHC (MHC).

Ontology	Upregulated genes	Downregulated genes
**Innate immunity**	**C1qb**, **C4b[MHC]**, **Chi3l3**, **Chi3l4**, **Cyba**, **Gvin1**, **Ifi205**, **Ifi47[CD]**, **Ifit3**, Map3k6, **Nlrc5**, Oasl1, **Oasl2**, Pglyrp1, Saa3, **Samd9l**, Serping1, **Trim21**	C1qtnf4
**Response to virus**	**Bcl2a1b**, **Bst2**, Eif2ak2, **Ier3[MHC]**, Ifi27l2a, **Ifit2**, **Ifitm1**, **Ifitm3**, **Isg15**, Ly6a, **Mx2**, **Oas1g[D]**, **Rsad2**, **Samhd1**	Skiv2l[MHC]
**Chemokines, cytokines, receptors**	**Ccl4**, **Ccl5**, **Ccl7[UC]**, **Ccl12[IBD]**, **Cxcl9**, **Cxcl10**, Osmr, **Socs3[MS,IBD,D]**	Cxcl12
**Response to stimulus, signal transduction**	**Angptl4**, **Ctsc**, **Fpr2**, Mt2, **Serpina3f**,	Acvr2b, Dgkb, Dgkz, Dlgap1, Grm4, Lphn1, **Mtss1l**, Pacsin1[MHC], **Prrt1[MHC, IBD]**, Psd2, Rasgef1a, Rgs7bp, Tnrc6a, Unc13c
**Adaptive Immunity, antigen processing and presentation**	**B2m**, **Cd274**, **Cd52**, **Cd74**, **Fcer1g**, Fcgr3[SLE,IBD], **Fcgr4[SLE, IBD]**, **H2-Ab1[C,MHC]**, **H2-D1/L[MHC]**, H2-Eb1[MHC, MS,RA], **H2-K1[MHC]**, **H2-K2[MHC]**, **H2-Q2[MHC]**, **H2-Q7[MHC]**, H2-Qa1[MHC], **H2-T22[MHC]**, **Psmb8[MHC]**, **Psmb9[MHC]**, **Tap1[MHC]**, **Tap2[MHC]**	Fclrs
**Transcription factor, regulation of transcription**	**Batf2**, **D14Ertd668e**, **Irf1[UC]**, **Irf7[SLE]**, **Irf9**, Stat1[IBD], **Txnip**	2210018M11Rik, Arid1a, Atxn7l3, Atxn7l3b, Bcl11b, Carm1, E2f6, Foxq1, Gtf3a, Hes5, Hist1h2bf, Hopx, **Jhdm1d**, Klf7, Msl1, Myt1l, Ncoa1, Nfix[C], Pbrm1, **Prkcb[IBD]**, **Rbfox1**, Rora, Tcf4, Usf2, Zbtb44, Zbtb7a, **Zfp523**
**GTP signaling**	**Gbp2**, **Gbp3**, **Igtp**, **Irgm1[IBD]**, **Irgm2**	Gdi1, Gna11[MHC], Gnao1, **Rab14**, Rab5b, Rab6, **Rhobtb2**, **Rnd2**, Sept3, Tbc1d17
**Cell cycle and proliferation, cellular differentiation**	Arpc1b, Cdkn1a, Cenpa, Emp1, **Gh**, Prl, Tagln2, **Xdh**	**Arl8b**, Efna5, Elavl3, **Gm16517**, Itm2a, Ltbp4, Mau2, Mzt1, **Nckap1**, Nfib, Ntrk3, Ptn, **Rnf167**, Scrib, Sema6d, Strbp, Thra, Tmod1, Tob1
**Adhesion**	**Icam1[P,MS,IBD]**, Itgad, Lgals3bp, Lgals9[CD]	Bcan, **Cd47**, Celsr2, Ntm
**Apoptosis**	**Bcl2a1d**, Ifi27l1, **Serpina3g**, **Tspo**, **Xaf1**	Ank2, **Tia1**
**Protein kinase, phosphotase**	**Cmpk2**	**Akap1**, **Camkk2**, Dusp8, Fjx1, Kalrn, Mark2, Pak3, Ppp1r35, Ppp5c, Ptprd, Taok1
**Ubiquitination**	**Parp14**, **Rnf213**, **Trim25**, **Ubd[MHC]**, **Ube1l**, **Usp18**	Fbxo41, Usp11
**RNA processing, translation**		Brunol4, **Eif5a**, Mettl17, Pabpn1, Rbfox1
**Transport**	**Slc15a3**	**Abcf2**, Apba2, Arf5, **Cacng3**, Gabrb1, Gga3, Gria2, Hcn3, **Pea15a**, **Pltp**, Scamp3[7], Slc38a2, Slc38a5, Slc38a9, Slc40a1, Slco1c1, Syt4, **Tmco3**, Ugt8a
**Blood cells and vessels**	**Anxa2**, **Tgm2**	Alas2, **Ccm2**, Flt1, Hba-a1, Hbb-b1, Pak1, **Ppap2b**
**Neuronal and junctions**	**Mobp**	Epn2, **Gjc2**, Kif3a, Klc1, Palm, Shank3, **Spnb4**
**Metabolic processes**	Adamts9, Ch25h, Fkbp5[MHC], **Lyz1**, Phyhd1, Pnpla2, Sult1a1, **Ugt1a6a**, **Upp1**	0610007P14Rik, 1190002N15Rik, **Acot7**, Atp6v0d1, **B4galt3**, Cyp46a1, Glg1, Hsd3b2, Mbtps1, **Mgat4b**, Mus81, **Oxct1**, Pcyt2, Pde6d, Phldb1, Ppp1ca, **Sdr39u1**, Smpd1
**Biological processes or unannotated**	2410039M03Rik, 8430408G22Rik, **Glipr2**, **Gm12250**, Ms4a6d, Plac8, Plin4	1110012J17Rik, 2600009P04Rik, 2900011O08Rik, 3110047P20Rik, 4930402H24Rik, 6330407J23Rik, AI593442, Caln1, Cops7a, D14Abble, **D17Wsu92e**, E330009J07Rik, **Fam126b**, Fam171b, Fam178a, **Fam63b**, Fndc5, Gats, Jph4, Klhdc1, Lonrf2, Orf61, Rtn1, Sgtb, Tmem63b, Tspan3, Zfp385b, Zfp612
